# Defining a diverse core collection of the Colombian Central Collection of potatoes: a tool to advance research and breeding

**DOI:** 10.3389/fpls.2023.1046400

**Published:** 2023-04-26

**Authors:** Norma Constanza Manrique-Carpintero, Jhon A. Berdugo-Cely, Ivania Cerón-Souza, Zahara Lasso-Paredes, Paula H. Reyes-Herrera, Roxana Yockteng

**Affiliations:** ^1^ Corporación Colombiana de Investigación Agropecuaria-AGROSAVIA, Centro de Investigación Tibaitatá, Mosquera, Colombia; ^2^ Corporación Colombiana de Investigación Agropecuaria-AGROSAVIA, Centro de Investigación Turipaná, Montería, Colombia; ^3^ Institut de Systématique, Evolution, Biodiversité-UMR-CNRS 7205, National Museum of Natural History, Paris, France

**Keywords:** genetic diversity, molecular markers, population structure, mini-core collection, polyploid

## Abstract

The highly diverse Colombian Central Collection (CCC) of cultivated potatoes is the most important source of genetic variation for breeding and the agricultural development of this staple crop in Colombia. Potato is the primary source of income for more than 100.000 farming families in Colombia. However, biotic and abiotic challenges limit crop production. Furthermore, climate change, food security, and malnutrition constraints call for adaptive crop development to be urgently addressed. The clonal CCC of potatoes contains 1,255 accessions ― an extensive collection size that limits its optimal assessment and use. Our study evaluated different collection sizes from the whole clonal collection to define the best core collection that captures the total genetic diversity of this unique collection, to support a characterization more cost-effectively. Initially, we genotyped 1,141 accessions from the clonal collection and 20 breeding lines using 3,586 genome-wide polymorphic markers to study CCC’s genetic diversity. The analysis of molecular variance confirmed the CCC’s diversity with a significant population structure (Phi=0.359; *p-value*=0.001). Three main genetic pools were identified within this collection (CCC_Group_A, CCC_Group_B1, and CCC_Group_B2), and the commercial varieties were located across the pools. The ploidy level was the main driver of pool identification, followed by a robust representation of accessions from Phureja and Andigenum cultivar groups based on former taxonomic classifications. We also found divergent heterozygosity values within genetic groups, with greater diversity in genetic groups with tetraploids (CCC_Group_B1: 0.37, and CCC_Group_B2: 0.53) than in diploid accessions (CCC_Group_A: 0.14). We subsequently generated one mini-core collection size of 3 percent (39 entries) and three further core collections sizes of 10, 15, and 20 percent (i.e., 129, 194, and 258 entries, respectively) from the total samples genotyped. As our results indicated that genetic diversity was similar across the sampled core collection sizes compared to the main collection, we selected the smallest core collection size of 10 percent. We expect this 10 percent core collection to be an optimal tool for discovering and evaluating functional diversity in the genebank to advance potato breeding and agricultural-related studies. This study also lays the foundations for continued CCC curation by evaluating duplicity and admixing between accessions, completing the digitalization of data, and ploidy determination using chloroplast count.

## Introduction

Crop diversity is the primary source of genetic variation for crop improvement and the development of cultivated species. Potato (*Solanum tuberosum* L.) is a species with great diversity in the primary gene pool. As domestication took place, several cultivated species co-evolved, from the most ancient cultivated potato, *S. stenotomum*, to the modern and most globally commercialized potato (Spooner et al., 2014). Based on the latest taxonomic classification, there are four cultivated potato species, *Solanum tuberosum* L., with two cultivar groups, Andigenum and Chilotanum, *S*. *ajanhuiri*, *S. juzepczukii*, and *S. curtilobum* (Spooner et al., 2007). The Andigenum group collapsed several former species from the previously accepted Hawkes (1990) classification, combining *S. stenotomum* Juz. & Bukasov, *S. phureja* Juz. & Bukasov, *S. chaucha* Juz. & Bukasov, and *S. tuberosum* subsp. *andigenum* Hawkes. Various dynamic and evolutionary factors influencing the traditional potato cropping of Andean communities generated the broad genetic diversity of cultivated potatoes. Those factors are a continuous hybridization of cultivated diversity mediated by the outcrossing nature of most diploids, the production of unreduced gametes, the presence of cultivated diversity with different ploidy levels in the same field, simultaneous sexual and asexual reproduction of which vegetative propagation is the main system, and possible crosses with wild germplasm growing close to cultivated fields. All of these factors promoted the generation and maintenance of the enormous variation of cultivated potato varieties over time (Dodds and Paxman, 1962; Huamán and Spooner, 2002; Spooner et al., 2010).

Potato landraces originated in South America; most of them are from the highlands of the Andes (found at 3000 − 4000 m of elevation), from western Venezuela to the south in southern Bolivia and northern Argentina, except for the Chilotanum group landraces found in the lowlands of the central and south-central area of Chile (Spooner et al., 2010). The landraces grown in Central America and Mexico came from Post-Columbian introductions. Based on the analysis of the eco-geographical distribution of potato landraces, the *S. tuberosum* Andigenum group has the most distant geographic coverage, with the tetraploids most widespread, followed by diploid and triploid ploidy levels. Like the Chilotanum group, *S. ajanhuiri*, *S. curtilobum*, and *S. juzepczukii* have a much smaller distribution, restricted to central Peru and Bolivia. From this ample distribution, several thousand potato landraces have been reported with broad genetic diversity that can be described at various levels: morphological, physiological, and agronomic are just some to be highlighted. Some authors have reported extensive diversity in tuber skin and flesh color, tuber and leaf shape, floral colors, growth habit, maturity, dormancy, and photoperiod length needed for tuberization, in addition to pest and disease resistance, environmental responsiveness, and tuber yield (Spooner et al., 2010; Ellis et al., 2020; Bradshaw, 2021). This extensive diversity represents a reservoir of genes for crop improvement, food security, and adaptability to climate change (ibid).

Colombia is considered a center of cultivated potato diversity, as it is one of the Andean countries where the potato was domesticated. Potato is an important staple for food security in Colombia and the primary source of income for around 100.000 farming families (Ministerio de Agricultura y Desarrollo Rural, 2020). Potato diversity in Colombia is conserved *in situ* and *ex situ*. The *in situ* conservation depends on some isolated indigenous communities and smallholder farmers who still conserve traditional landraces (Tinjacá and Rodríguez, 2015). This is because potatoes cultivation in most of the territory uses modern commercial practices. On the other hand, *ex situ* potato conservation is carried out by the Colombian Corporation for Agricultural Research-AGROSAVIA (Corporación Colombiana de Investigación Agropecuaria in Spanish) and universities such as Universidad Nacional de Colombia, among others, using three different systems: seeds, field, and *in vitro*. Farmers and private companies also try to conserve and promote the commercialization of native potatoes. The Colombian Central Collection (CCC) of potato was started in the 1950s as the basis for a genetic platform for potato research and breeding initiative promoted by several Colombian plant scientists, agronomists, and farmers, supported by the Agriculture Minister of Colombia, and guided by Dr. Jack Hawkes (Luján, 1976; Pineda Colorado and Hernández Castillo, 1996) who established the national potato breeding program. Today, AGROSAVIA manages the CCC and conserves 2,499 wild and cultivated potato accessions. The clonal collection of cultivated germplasm has 1,255 accessions, most of which belong to the *S. tuberosum* Andigenum group and a few accessions to the *S. tuberosum* Chilotanum group based on Spooner et al. (2014). However, in the CCC, the germplasm is classified as *S. phureja*, *S. tuberosum* subsp. *andigenum*, and *S. tuberosum* subsp. *tuberosum*, following Hawkes (1990) taxonomic classification.

The CCC diversity has been important for advancing research and breeding of potato in Colombia. The assessment of genetic diversity in part of the clonal CCC showed that it is highly diverse, with high and significant population structure and greater percentage of genetic variation within than between populations (Juyó et al., 2015; Berdugo-Cely et al., 2017). This collection has been used as a source for late blight resistance caused by *Phytophthora infestans*, Guatemalan tuber moth *Tecia solanivora*, and drought stress (Santa Sepúlveda, 2018; Díaz-Valencia et al., 2021). These are some of the most limiting constraints of potato crop production. Moreover, the CCC of potatoes has been used for the generation and release of new varieties over the last 70 years in Colombia (Pineda Colorado and Hernández Castillo, 1996; Ñustez, 2011). Similarly, in 1995 AGROSAVIA scientists initiated the morphological characterization of the CCC, together with different evaluations of essential traits for breeding, such as late blight resistance, maturity, and quality attributes (Moreno and Valbuena, 2006; Berdugo-Cely et al., 2017). Most of the evaluations were done by groups of accessions over an extended timeline because of the collection size and limited resources at different levels, such as trained personnel, funding, infrastructure, etc. The potato crop in Colombia is subject to several biotic and abiotic challenges, besides food security, malnutrition, and climate-change constraints. Thus, the CCC has been and will remain the primary source of genetic diversity for crop improvement in Colombia.

The size of a collection, particularly extensive collections, could limit the possibility of fully documenting and using the germplasm because of the high logistics and infrastructure resources required to undertake research and evaluation of the collection. Thus, Frankel (1984) proposed rationalizing the collections by reducing redundancy and generating core collections, a representative subset of the whole collection diversity. In general, the purpose of a core collection is to maximize the representation of full genetic diversity. However, a core collection can also be assembled to capture the diversity in contrasting or extreme trait phenotypes, or to represent patterns of diversity based on different criteria such as geographic distribution, the proportion of diversity by clusters, and diversity of a trait, among others (Odong et al., 2013). In a core collection ― designed to represent the full diversity of the primary collection ― each selected accession or entry represents all the accessions in the entire collection that are similar to it. Thus, each entry in the core collection could represent one or more accessions in the entire collection. This is possible because the selection occurs from the center of unique weights or clusters that conform to a uniform representation across the whole diversity. Odong et al. (2013) recommended using genetic distance-based metrics to select entries in a core collection representing genetic diversity, precisely the minimal average distance between each Accession and the Nearest Entry (A–NE). Thus, to guarantee a good selection of a core collection, the value of the A–NE distance should be the smallest possible, considering that itself (A–NE=0) is the maximum representation of one accession. Since a genetic distance-based metric is the best criterion for generating a diverse core collection, molecular markers rather than phenotypic or passport data should be used to find a core collection that maximizes the representation of all genetic diversity in the main collection. Most molecular markers are selectively neutral, unaffected by the environment, and are more suitable for statistical genetic diversity analysis (Brown, 1989; Chapman and Crawford, 1990). Developing many markers with genome-wide coverage that ensure the best genetic diversity representation by the selected entries is straightforward today.

This study evaluated the CCC´s population structure and genetic diversity using genome-wide single nucleotide polymorphisms (SNPs). Similarly, we assembled a core and mini-core collection using the Average Accession to Nearest Entry (A-NE) method and validation recommended by Odong et al. (2013). The best core and mini-core collections were identified by comparing their genetic diversity measurements with the one in the whole CCC. Then, the quality and usefulness of the selected core collection was validated by comparing the phenotypic diversity representing the whole CCC with the core and mini core collections for three different agronomic traits (Average Tuber Weight in g (ATW), Number of Tubers per Plant (NTP), and Total Tuber Yield (TTY) in kg/plant). Finally, in this study, we set the basis to continue the CCC curation by evaluating duplicity and admixing between accessions, completing the characterization, data digitalization, and ploidy determination using chloroplast count.

## Materials and methods

### Plant material

For this study, we used 1,141 accessions from the field and *in vitro* copies taken from the clonal CCC of potatoes genebank managed by AGROSAVIA and 20 commercial varieties and advanced breeding lines obtained from different sources. This CCC contains mainly landraces and a small proportion of released varieties. Based on Spooner et al. (2007) taxonomy, most accessions belong to the species *S. tuberosum* group Andigenum, and few accessions to *S. tuberosum* group Chilotanum. However, the classification of the material in the potato collection follows Hawkes (1990) previously accepted taxonomic classification. According to this classification, 143 accessions are *S. phureja*, 681 *S. tuberosum* subsp. *andigenum*, 67 *S. tuberosum* subsp. *tuberosum*, one (1) *S. rybinii* (older classification), while for 269 accessions we currently do not have the information because it is in the curation process and digitalization of data from hard copy records. Based on the original classification, this study named the accessions Andigena, Phureja, and Tuberosum types. The geographic origin of the accessions is diverse; we have records for 802 accessions, from which 670 are from Colombia, 95 from other Latin American countries, 26 from Europe, and 11 from the United States (**Table 1**).

**Table 1:** Geographic origin of accessions held in the Colombian Central Collection of potatoes conserved at the Corporación Colombiana de Investigación Agropecuaria (AGROSAVIA).

Country of origin	Number of accessions
Argentina	1
Belgium	1
Bolivia	14
Brazil	1
Colombia	670
Ecuador	13
Germany	2
Netherlands	11
Mexico	5
Perú	60
Scotland	4
United Kingdom	8
United States	11
Venezuela	1
Total	802

### Sampling and genotyping

We collected leaf tissue samples for each accession from 1−2 month-old *in vitro* plantlets or field grown plants at vegetative stage. In the latter case, the tissue was collected from one randomly selected plant out of the 20 clones grown per accession. The CCC potato curators regenerate the field collection annually in a highland area of Zipaquirá municipality, department of Cundinamarca, Colombia, at 2.950 m altitude, with an average temperature of 15°C and relative humidity of 75 percent. We extracted DNA from the leaf tissue using the DNeasy Plant Mini Kit (Qiagen, Valencia, CA, USA), following the manufacturer’s technical recommendations. Then, we genotyped each accession with the Illumina single nucleotide polymorphism (SNP) Infinium Potato Array version 8303 (Felcher et al., 2012) and scanned the fluorescent signals in the Illumina HiScan SQ system (Illumina, San Diego, CA) at AGROSAVIA. A set of 809 samples already had a genotyping profile processed by Berdugo-Cely et al. (2017). Therefore, we genotyped 482 additional samples to obtain a total of 1,291 samples genotyped and used in this study (**Supplementary Table S1**). Of these samples, 1,061 are unique accessions and 130 are biological repetitions. The accessions in the clonal CCC did not have unique identification numbers, and samples were collected from field and *in vitro* sources using another nomenclature. As part of the CCC curation process, the unique identifier numbers were assigned during this genotyping process; this allowed us to identify several samples that had been genotyped multiple times. Due to limitations in budget, samples were genotyped in batches in order to complete the samples evaluated in this study, which contributed to accumulating several duplicates in the genotyping process. We obtained the raw values from the fluorescent signals for all samples using GenomeStudio 2.0 software (Illumina, San Diego, CA). Clustercall package (Schmitz Carley et al., 2017) in the R platform (R Core Team, 2021) transformed these signal values to obtain the tetraploid genotype calls (AAAA, AAAB, AABB, ABBB, BBBB coded as 0, 1, 2, 3, 4).

### Population structure and genetic diversity

The final SNP genotype matrix was obtained from the ClusterCall output matrix, filtered to remove markers with minor allele frequency of less than 0.03 and missing data greater than 5 percent at the genotype and marker level. We assessed population structure using this dataset with tetraploid genotype calls through two strategies: i) a Bayesian model implemented in the software STRUCTURE 2.3 (Pritchard et al., 2000) without *a priori* population information, and ii) a Principal Component Analysis (PCA) calculated using the R-Adegenet package (Jombart and Ahmed, 2011). For the Bayesian model, the number of populations (in this study referred to as genetic groups) K was estimated from one to ten using an admixture model with frequencies of correlated alleles, a burn-in set to 150.000 interactions, and ten independent repetitions per run. Then, we found the best number of populations in the Structure Harvester program (Earl and vonHoldt, 2012) using the Evanno et al. method (Evanno et al., 2005). The NbClust R-package (Charrad et al., 2014) was used to determine the K for the PCA loading as input the information of the first three components. We tested if there was population differentiation by calculating the F_ST_ and the percentages of differentiation between and within identified subpopulations using the analysis of molecular variance (AMOVA) in the R-packages dartR (Mijangos et al., 2022) and Poppr (Kamvar et al., 2014), respectively. Finally, we manually estimated the genetic diversity by calculating the observed heterozygosity (Ho) per genotype and genetic group, using the methodology described by Berdugo-Cely et al. (2021).

### Ploidy-level prediction

We predicted the ploidy level of each accession using the proportion of simplex and triplex genotype scores (ABBB, AAAB) across all SNPs per accession, according to Alsahlany et al. (2019). This method sets up the parameters to predict the ploidy using a set of reference samples. In that case, when the sum of simplex and triplex proportions was close to zero, the sample would be diploid, and when the frequency was greater than 0.2 it would be tetraploid. In this study, we validated the accuracy of this method by comparing the ploidy of 112 reference accessions from the CCC assessed with SNP genotype score frequencies and the ploidy values obtained using chloroplast and chromosome counts from three publications (Guevara, 2011; Uribe Gaviria, 2011; Sánchez, 2017). For this analysis, we implemented a one-way analysis of variance (ANOVA) and Tukey test in R software to find significant differences among the ploidy prediction strategies. Finally, the parameters were established to assess the ploidy in the samples used in this study.

### Core collections

We used the Core Hunter 3 program (de Beukelaer et al., 2018) to define core collections representing the total genetic diversity present in the CCC of potatoes. The Modified Roger’s genetic distances (MRD) were calculated and used to identify the core sets with the A–NE distance function. This function optimizes the entry selection to minimize the average distance between each accession in the whole collection and the closest-selected entry, as expected when creating a diverse core collection. We tested three collection sizes of 10, 15, and 20 percent of the total samples evaluated in this study (i.e., 129, 194, and 258 entries, respectively) and one mini-core collection with 3 percent of the original size (39 entries).

We assessed the quality of the A-NE method for core collection selection by calculating and comparing the average criterion distance for A-NE and two additional distance-based metrics evaluated by Odong et al. (2013) for each of the core and mini-core collections identified: (1) average distance between each entry and the nearest neighboring entry (E-NE), and (2) average genetic distances between entries (E-E). The EvaluateCore R package v.0.1.3 (Aravind et al., 2022) and two additional core sizes (50 and 80 percent) were used for this comparison.

To validate that the genetic diversity representation was kept across collections and select the most suitable core collection for breeding and research, we compared the average observed heterozygosity (Ho) measurements from the core and mini-core collections to the average in the whole CCC. Additionally, a PCA was carried out for each collection to show the distribution of the samples selected in each proposed core collection compared to the whole CCC. Finally, for the 3 percent mini-core collection identified using Core Hunter, we replaced some selected entries to match and contain all accessions within the 10 percent core collection. For this, we identified the entries that differed between the 3 percent mini-core and 10 percent core collections from the initial Core Hunter entry selection. Then, using the MRD matrix, we calculated the difference between the genetic distance of each entry identified in the 3 percent mini-core and the 10 percent entries to identify the 10 percent entry with the minor genetic distance difference. Finally, the 3 percent entries were replaced with the corresponding 10 percent core entry identified, which were instead selected for 19 accessions.

### Validation of core and mini-core collections’ usefulness

We validated the effectiveness of the accessions in the core and mini-core collections to represent the whole spectrum of phenotypic variation for three different traits: i) Average Tuber Weight (ATW) in grams, ii) Number of Tubers per Plant (NTP), and iii) Total Tuber Yield (TTY) in kg/plant. For this we used the phenotypic data of 846 genotyped accessions (206 for CCC_Group_A, 494 for CCC_Group_B1, and 146 for CCC_Group_B2) for which preliminary field evaluations had been conducted for ATW, NTP, and TTY. Data were collected between 2013 and 2015. Accession data collected across these years depends on the accessions evaluated during each field campaign. Due to financial and operational capacity limitations, we prioritized accessions and traits to be evaluated by year. Andigenum and later the Phureja accessions were evaluated in three consecutive years. The evaluations were conducted in a highland area of Zipaquirá municipality, in the department of Cundinamarca. Field data for each year was collected from an average of 20 clones planted in one tier per accession, using a complete randomized design for the entire CCC in the field. Thus, each year’s data represents a repetition. We used the mean values from all-year evaluations per trait and accession for the analyzes. We expected that, independently of the size, a good core collection would show a similar phenotypic distribution as the one for the 846 accessions. Moreover, we analyzed to see if the identified entries for the different core and mini-core collections represented the phenotypic diversity of all accessions by comparing their average criterium distance for A-NE, E-NE, and E-E metrics. The Gower distance (Pavoine et al., 2009) between accessions was calculated using the phenotypic variable matrix. Then, like in the above evaluation for SNPs, the EvaluateCore R program was loaded with this information to calculate the different distance-based metrics. We used selected entries in the core and mini-core collections that contained phenotypic data.

## Results

### Population structure and genetic diversity

**Figure 1:**
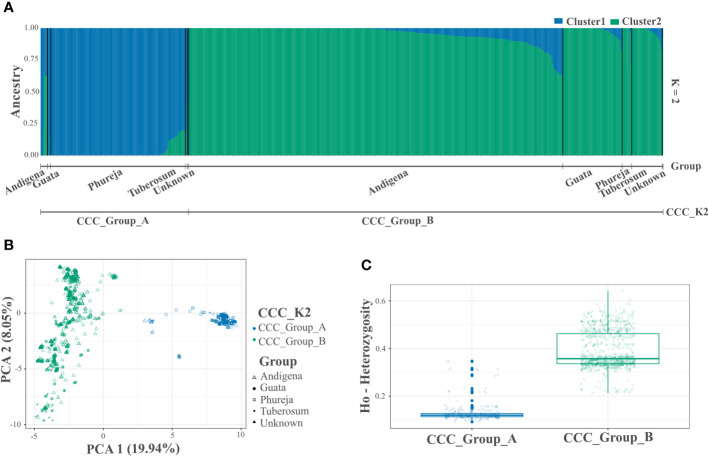
Population structure and genetic diversity analyzes of the Colombian Central Collection (CCC) of potatoes for two genetic groups (CCC_K2). **(A)** Bar plot of genetic structure analysis for two genetic groups (K=2) identified using the STRUCTURE software and designated as CCC_Group_A and CCC_Group_B (lower line). The AGROSAVIA in-house classification (Group) is indicated in the upper line (Andigena, Phureja, Tuberosum, “Guata” and Unknown), **(B)** Scatter plot of the Principal Component Analysis (PCA) color coded by genetic group and shaped following AGROSAVIA in-house biological status classification (Group), and **(C)** Box plot with distribution (box representing the second and third quartiles, line across the median, and vertical line the range of data) with distribution of observed heterozygosity (Ho) for each genetic group.

**Figure 2:**
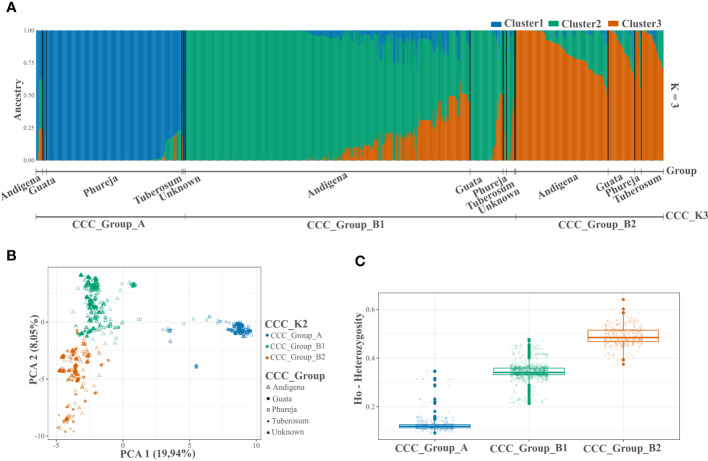
Population structure and genetic diversity analyzes of the Colombian Central Collection (CCC) of potatoes for three genetic groups (CCC_K3). **(A)** Bar plot of genetic structure analysis for three genetic groups (K=3) identified using the NbClust R-package and designated as CCC_Group_A, CCC_Group_B1, and CCC_Group_B2 (lower line). The AGROSAVIA in-house biological status germplasm classification (CCC_Group) is indicated in the upper line (Andigena, Phureja, Tuberosum, “Guata” and Unknown), **(B)** Scatter plot of the Principal Component Analysis (PCA) color coded by genetic group and shaped following AGROSAVIA in-house biological status classification (CCC_Group), and **(C)** Box plot with distribution (box representing the second and third quartiles; line across the median, and vertical line representing the range of data) with distribution of observed heterozygosity (Ho) for each genetic group.

After quality controls and filters, we identified 3,586 genome-wide SNP markers to conduct the analyzes (**Supplementary Table S2**). The population structure analysis using the STRUCTURE and NbClust approaches suggested two and three genetic groups within the CCC germplasm, respectively (**Figures 1A, B**, **2A, B** and **Supplementary Figures S1A, B**). The ploidy level was the main driver of pool identification, followed by the taxonomic classification initially assigned to the different accessions in the collection (**Table 2**). The population genetic analyzes supported these results, showing a significant solid genetic structure for the two (Phi=0.370; *p-value*=0.001) and three (Phi=0.359; *p-value*=0.001) genetic groups detected in the CCC. The PCA also showed similar results (**Figures 1B**, **2B**). The first and second components explained 19.94 percent and 8.04 percent of the sample variance, endorsing the differentiation of accessions by ploidy level and taxonomy with some level of admixture.

**Table 2:** Taxonomic and ploidy characteristics of three genetic groups identified in the Colombian Central Collection (CCC) of potatoes, based on population structure and genetic diversity analyzes.

Genetic group	Taxonomic classification	Ploidy*			Total	Percentage
		2*x*=2n=24	2*x*=4n=48	Unknown		
**CCC_Group_A**		239	9	–	248	21.3%
	*S. rybinii*	1	–	–	1	0.4%
	*S. tuberosum *subsp. *andigenum*	5	2	–	7	2.8%
	*S. tuberosum* subsp.*phureja*	136	1	–	137	55.2%
	*S. tuberosum* subsp. *tuberosum*	4	–	–	4	1.6%
	*S. tuberosum* sp. (In the curation process)	93	6	–	99	39.9%
**CCC_Group_B1**		–	619	21	640	55.1%
	*S. tuberosum* subsp. *andigenum*	–	505	16	521	81.4%
	*S. tuberosum* subsp. *phureja*	–	2	–	2	0.3%
	*S. tuberosum *subsp.* tuberosum*	–	16	1	17	2.7%
	*S. tuberosum* sp. (In the curation process)	–	96	4	100	15.6%
**CCC_Group_B2**		–	273	–	273	23.5
	*S. tuberosum *subsp. *andigenum*	–	153	–	153	56.0%
	*S. tuberosum* subsp. *phureja*	–	4	–	4	1.5%
	*S. tuberosum* subsp. *tuberosum*	–	46	–	46	16.8%
	*S. tuberosum* sp. (In the curation process)	–	70	–	70	25.6%
s **Total**		239	901	21	1161	100%

*Ploidy assessment base on the frequency of simplex and triplex Single Nucleotide Polymorphism (SNP) marker scores per accession.

In the latest taxonomic classification, the most traditional and modern cultivated potatoes are classified within a single potato species. However, the Hawkes taxonomy originally assigned to the CCC aligns with a clear genetic differentiation inherent to the germplasm. Thus, for this paper, we designated the accessions as Phureja, Andigenum, and Tuberosum types. The population structure analysis conducted using STRUCTURE discriminated the CCC into two genetic groups, the CCC_Group_A with mainly diploid (2n=2*x*=24) and CCC_Group_B with mostly tetraploid (2n=4*x*=48) accessions. The second analysis conducted using Nbclust detected three genetic groups, the same diploid cluster (CCC_Group_A) previously identified, while the tetraploid cluster was separated into two new genetic groups (CCC_Group_B1 and CCC_Group_B2). As shown in **Table 2**, the CCC_Group_A genetic group contained 248 accessions. Of these, 96.4 percent (n=239) were diploids, and 3.6 percent (n=9) were tetraploids. At the taxonomic level, most of the accessions (n=137, 55.2 percent) were Phureja type, while 2.8 percent (n=7) Andigenum, 1.6 percent (n=4) Tuberosum, 0.4 percent (n=1) was *S. rybinni*, and for 39.9 percent (n=99) we have no information. There has been no digitalization of hard copy records of the taxonomic classification assigned to these accessions when the CCC material was acquired. Therefore, this documentation is currently in the process of curation. The CCC_Group_B1 had 640 accessions, 96.7 percent (n=619) tetraploid accessions, and 3.2 percent (n=21) with unknown ploidy. Taxonomically, most of the accessions, 81.4 percent (n=521), were Andigenum, and a lower proportion Tuberosum, 2.7 percent (n=17), and Phureja, 0.3 percent (n=2). The remaining 15.6 percent (n=100) are currently undergoing taxonomic classification. Finally, the CCC_Group_B2 genetic group had 100 percent (n=273) tetraploid accessions. At the taxonomic level, 56 percent (n=153) corresponded to Andigenum, 16.8 percent (n=46) Tuberosum, 1.5 percent (n=4) Phureja, and 25.6 percent (n=70) are pending taxonomic classification. Finally, the accessions evaluated in this study that are released varieties or breeding program germplasm ― some of which are part of the CCC ― were evenly located across genetic groups. Accordingly, some were diploids and other tetraploids.

The genetic differentiation is significant between the CCC_Group_A and the CCC_Group_B genetic groups (F_ST_=0.146, *p-value*<0.0001). The CCC_Group_A shows lower genetic differentiation with CCC_Group_B1 than with CCC_Group_B2 genetic groups (F_ST_=0.164, *p-value*<0.0001 and 0.206, *p-value*<0.0001 respectively), while the tetraploid groups are more similar (F_ST_=0.071, *p-value*<0.0001). Thus, the CCC_Group_B1 has an intermedium differentiation level between CCC_Group_A and CCC_Group_B2 genetic groups. The results show high genetic variation in the CCC germplasm, confirmed by a higher significant variation within genetic groups [62.95 percent for genetic structure with two groups (K2) and 64.08 percent for three groups (K3)] than between groups (37.04 percent for K2 and 35.91 percent for K3). Based on divergent heterozygosity values (Ho) for the genetic structure of two (CCC_Group_A: 0.14 ± 0.04 and CCC_Group_B: 0.42 ± 0.08) and three genetic groups (CCC_Group_A: 0.14 ± 0.04; CCC_Group_B1: 0.37 ± 0.04, and CCC_Group_B2: 0.53 ± 0.04), the genetic variation within the CCC_Group_B, B2, and B1 groups was greater than that observed in CCC_Group_A group (**Figures 1C**, **2C**).

### Ploidy assessment

**Figure 3:**
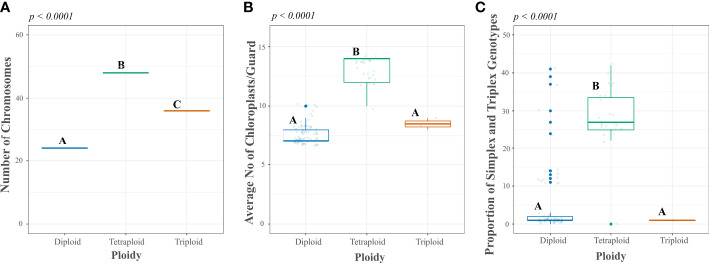
Comparison of direct and indirect methods of ploidy assessment in a reference set of accessions from the Colombian Central Collection (CCC) of potatoes. Y-axes show the measurement scales of direct and indirect methods: **(A)** chromosome count in the root tips, **(B)** chloroplast count per guard cell, and **(C)** proportion of simplex and triplex (ABBB, AAAB) Single Nucleotide Polymorphic (SNP) genotype scores per sample, compared to the x-axes with the ploidy base on the direct method of ploidy assessment. The *p-values* correspond to the ANOVA significance, while the different letters correspond to test results using a Tukey test in R software.

We used a reference sample of 112 accessions to confirm the accuracy of predicting the ploidy level of the CCC accessions using the proportion of simplex and triplex SNP marker scores per sample, according to Alsahlany et al. (2019). This assessment was compared to another indirect ploidy assessment method, the average number of chloroplasts per guard cell, and ― a direct approach ― the number of chromosomes counted in root cells from previously published data (Guevara, 2011; Uribe Gaviria, 2011; Sánchez, 2017). In general, the reference set of samples showed a pattern for the number of chloroplasts per guard cell and the proportion of simplex and triplex scores of all SNPs per sample associated with the number of chromosomes (**Figure 3** and **Supplementary Table S1**). The ploidy level based on chromosome count, the direct and precise method of ploidy assessment, for the 112 accessions in the reference set included 83 diploids (2n=2*x*=24), 27 tetraploids (2n=4*x*=48), and two triploids (2n=3*x*=36). As expected, the *Solanum* genus’ primary number of chromosomes (12) was duplicated 2−4 times according to each accession’s ploidy level (**Figure 3A** and **Supplementary Table S1**). Thus, this ploidy assessment was used to compare the accuracy of the indirect methods. The chloroplast counts in guard cells matched an average of 7.6 ± 0.83 chloroplasts/guard cells for diploid accessions and 13.2 ± 1.06 chloroplasts/guard cells for tetraploid accessions. The tetraploid group differed significantly from the diploid group (*p-value <*0.0001). The chloroplast count in guard cells could not predict triploid accessions. Most diploid accessions (74/83, 89.2 percent) had 7−8 chloroplasts/guard cell average, and a few (9/83, 10.8 percent) were in a grey area between 9−10 chloroplasts/guard cell average. The tetraploids were more consistent with 12−14 chloroplast/guard cells for 96 percent of accessions (26/27) and one in a grey area of 10 chloroplasts/guard cells.

When using the proportion of SNP genotype scores as an indirect method for assessing ploidy, this analysis showed an average of 4.8 percent ±9.04 simplex and triplex SNP genotype scores for diploid accessions and a 27.1 percent ±1.53 for tetraploid accessions. Moreover, the ANOVA confirmed significant differences between the average for diploid and tetraploid samples (*p-value* < 0.001), as shown in **Figure 3C** and **Supplementary Table S1**. Similarly to the chloroplast count method, this method could not discriminate triploid samples. From the total diploid accessions based on chromosome count, for 77 the proportion of simplex and triplex SNP scores was 0−15 percent, and for six accessions the frequency of simplex and triplex SNP scores was off-type with 24−41 percent. For the tetraploid samples, the proportion of simplex and triplex SNP genotype scores per accession was accurate for 26 tetraploid samples with values of 22−42 percent and one sample off-type with 0 percent. The mismatch between methods could be due to sample admixture between the first and current evaluations since the sampling for each assessment occurred at separate times. Unlike for chloroplast and chromosome counts, this mismatch did not occur, and the methods were assessed using samples from the same source of plant materials. If we consider them real method errors, ploidy prediction accuracy using simplex and triplex SNP genotype scores per accession was 92.8 percent (77/83) for diploids and 96.3 percent for tetraploids (26/27).

Based on these results, we predicted the ploidy of all samples used in this study using a threshold of 0−15 percent frequency of simplex and triplex SNP genotype scores per accession for diploids and greater than 20 percent for tetraploids. The unknown ploidy corresponded to accessions with a frequency of simplex and triplex in a grey area of 15−20 percent. Of the 1,291 genotyped samples, 239 unique accessions were diploids, 901 tetraploids, and 21 unknowns (**Table 2**). The ploidy for the 130 biological repetitions included 59 diploids, 70 tetraploids, and one unknown. For 101 accessions with biological replicates, the ploidy assessment matched among repetitions, while for 12 accessions, some repetitions did not have the same results.

### Core collection assembly

**Figure 4:**
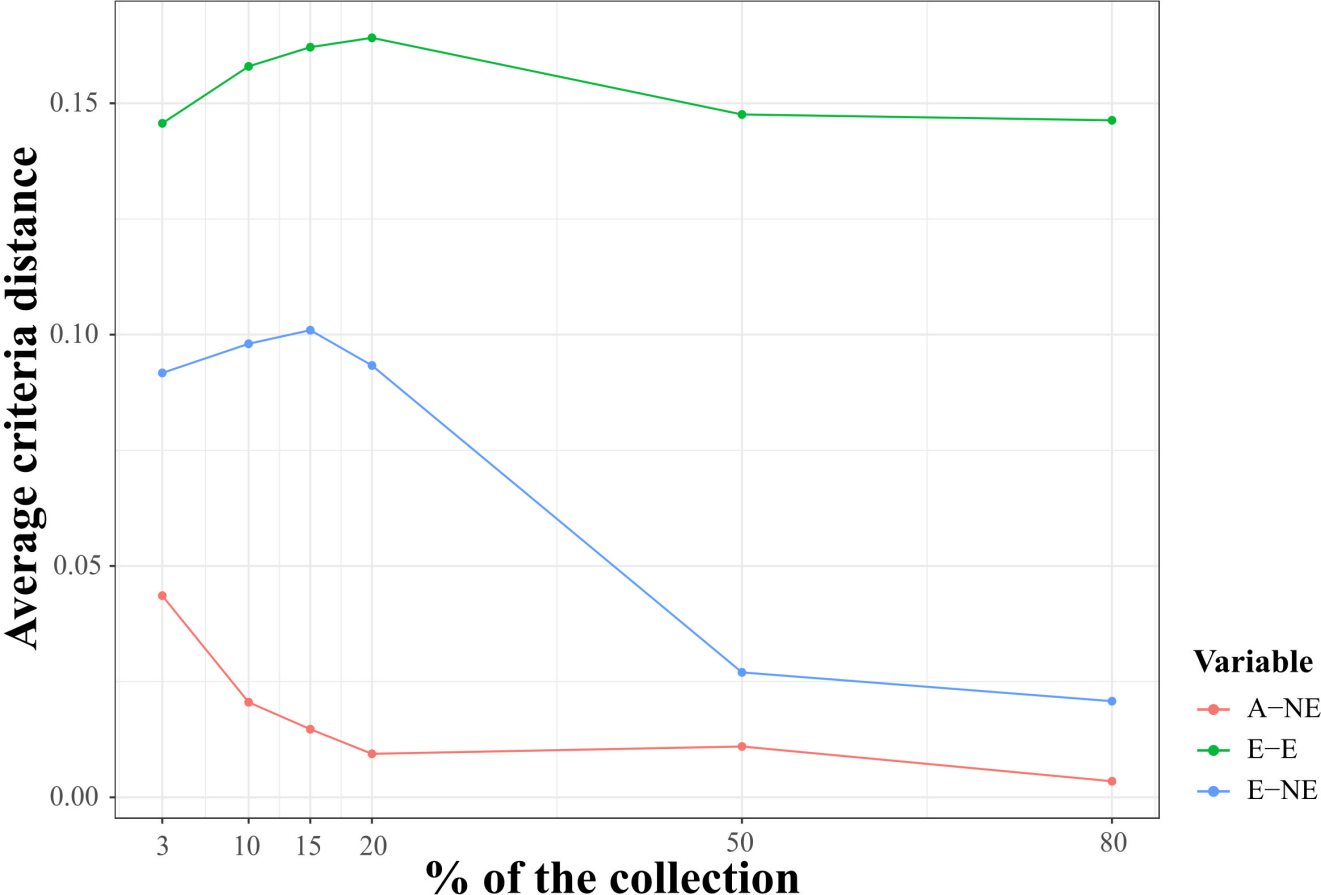
Qualitative evaluation of average Accession to Nearest Entry (A-NE) metrics used for diverse core collection identification and quality of core collections. The average criteria distance (y-axes) for three distance-based metrics A-NE, E-E (average Entry to Entry distance), and E-NE (average Entry to Neatest Entry distance) calculated using the core entries identified with A-NE criterion and single nucleotide (SNP) markers for six core collection sizes of 3, 10, 15, 20, 50, and 80 percent of whole samples genotyped in this study (x-axes).

**Figure 5:**
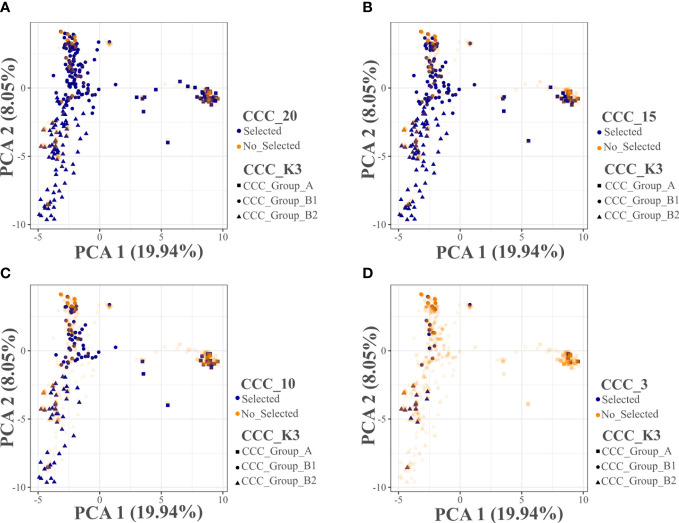
Principal Component Analyses (PCA) distribution of the selected entries in three core and one mini-core collections evaluated for the Colombian Central Collection (CCC) of potatoes. **(A)** Core collection size of 20 percent (CCC_20), **(B)** Core collection size of 15 percent (CCC_15), **(C)** Core collection size of 10 percent (CCC_10), and **(D)** Mini-core collection size of 3 percent (CCC_3). The CCC_K3 information in **(A–D)** corresponds to the three genetic groups suggested by the CCC population structure analysis using the NbCluster R-package.

**Figure 6:**
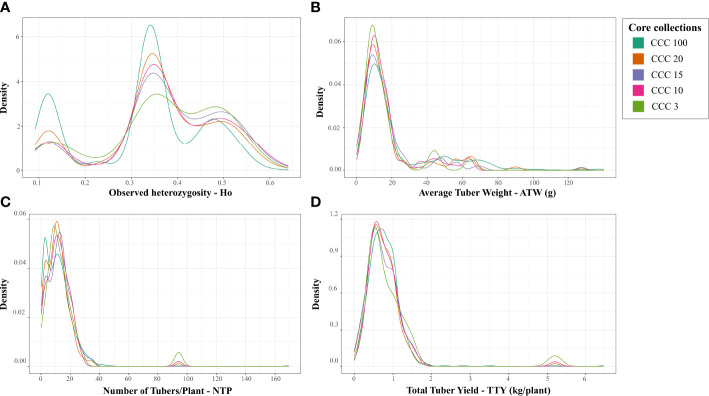
Density distribution of the genetic diversity and three different agronomic traits values of samples compared across main and proposed core and mini-core collections for the Colombian Central Collection (CCC) of potatoes. Distribution of values for **(A)** observed heterozygosity (Ho), **(B)** Average Tuber Weight in g (ATW), **(C)** Number of Tubers per Plant (NTP), and **(D)** Total Tuber Yield in kg/plant (TTY) for the whole CCC (CCC_100), and three core collection sizes of 20 percent (CCC_20), 15 percent (CCC_15), and 10 percent (CCC_10), and one mini-core collection size of 3 percent (CCC_3).

Using the Core Hunter 3 program, we identified three core collections for 10 (CCC_10), 15 (CCC_15), and 20 percent (CCC_20) of the total number of samples used in this study (i.e., 129, 194, and 258 entries, respectively), plus one mini-core collection with 3 percent (CCC_3) of the original size (39 entries). The accuracy of the A-NE algorithm used to identify diverse core collections was confirmed. Lower values of average A-NE distance were observed for the core and mini-core collections identified using the A-NE. In contrast, the same entries in the different core collections had an average distance near the optimum maximum values for the E-E or E-NE distance criteria (**Figure 4**). Similarly, as expected, the greater the collection size, the smaller the average A-NE distance, since each accession represents itself in the whole collection. Then, we compared the genetic diversity representation across collections identified using the A-NE method. We found no differences between the core and mini-core collections compared to the primary collection using the PCA and Ho for this comparison. The PCA showed that all the core and mini-core sizes represented a similar pattern of genetic diversity distribution (**Figures 5A–D**) comparable to the entire collection (**Figures 1B**, **2B**). For the whole collection (CCC_100), the Ho mean was 0.33 ( ± 0.13), 0.36 ( ± 0.12) for the 20 percent (CCC_20) core collection, 0.38 ( ± 0.12) for the 15 percent (CCC_15), and 0.38 ( ± 0.13) for the 10 percent (CCC_10) core collections, while the Ho for the 3 percent (CCC_3) mini-core collection was 0.37 ( ± 0.13). In general, Ho mean was greater in the core collections (Ho = 0.36-0.38) compared to the whole CCC (Ho = 0.33), and the Ho density distribution from accessions in each collection follow similar patterns to the CCC and were contained within it (**Figure 6A** and **Table 3**). Therefore, we propose a 10 percent core collection for research and breeding in AGROSAVIA. The 10 percent core collection is a suitable size that captures the total diversity of the CCC and has a manageable number of accessions for research and breeding projects. The sample proportion in the three genetic groups (CCC_Group_A, CCC_Group_B1, and CCC_Group_B2) was 24 percent, 53 percent, and 24 percent for all genotyped samples, and 15 percent, 52 percent, and 33 percent for the 10 percent core collection.

**Table 3:** Measures of central tendency (mean and median) and dispersion [coefficient of variation (CV), standard deviations (Std Dev), minimal (min) and maximum (Max) values] for the observed Heterozygosity (Ho), Average Tuber Weight (ATW), Number of Tubers per Plant (NTP), and Total Tuber Yield (TTY) traits evaluated in the Colombian Central Collection (CCC_100) of potatoes and Core Collections sizes of 20 percent (CCC_20), 15 percent (CCC_15), 10 percent (CCC_10) and 3 percent (CCC_3) of the CCC proposed in this study.

Core Collection	NGGD	NGPD	Measure	Ho	ATW in g	NTP	TTY in kg/plant
CCC_100	1291	846	CV	39.91	100.94	88.7	55.37
			Mean	0.33	23.04	11.64	0.77
			Std dev	0.13	23.26	10.21	0.43
			Median	0.34	13.65	10.32	0.73
			Min	0.09	0.17	0.35	0.0
			Max	0.64	140.0	169.69	6.47
CCC_20	258	172	CV	34.42	108.94	80.59	64.8
			Mean	0.36	18.27	11.59	0.73
			Std dev	0.12	19.91	9.34	0.47
			Median	0.35	11.25	10.58	0.67
			Min	0.09	0.17	0.48	0.02
			Max	0.64	127.5	94.23	5.19
CCC_15	194	115	CV	32.39	103.01	59.89	48.8
			Mean	0.38	16.91	12.51	0.73
			Std dev	0.12	17.42	7.49	0.35
			Median	0.37	12.07	12.21	0.69
			Min	0.1	0.5	0.82	0.08
			Max	0.64	127.5	34.48	1.77
CCC_10	129	81	CV	33.35	85.58	87.53	73.32
			Mean	0.38	16.82	12.9	0.8
			Std dev	0.13	14.4	11.29	0.59
			Median	0.38	16.82	12.9	0.8
			Min	0.1	1.45	0.82	0.1
			Max	0.64	64.5	94.23	5.19
CCC_3	39	26	CV	34.84	91.64	116.24	102.07
			Mean	0.37	15.54	14.99	0.92
			Std dev	0.13	14.24	17.43	0.94
			Median	0.37	15.54	14.99	0.92
			Min	0.12	3.67	1.24	0.21
			Max	0.56	64.5	94.23	5.19

NGGD, Number of Genotypes with Genotypic Data; NGPD, Number of Genotypes with Phenotypic Data.

Finally, we used the available phenotypic data of three agronomical traits (ATW, NTP, and TTY) for 846 genotyped accessions of the CCC to validate the representation of phenotypic variation in the core and mini-core collections. The validation strategy demonstrated that the 846 genotyped and phenotyped accessions had a similar distribution range of values for central and dispersion statistics in the whole collection (CCC_100) and across the different core (CCC_20-10) and mini-core (CCC_3) collections for the three different traits (**Figures 6B–D** and **Table 3**). In contrast to the values of genetic diversity in terms of Ho across collections, that showed a trend towards increasing from the whole collection to the smaller size of the core collection (**Figure 6A**), for the ATW trait, the means in the core collections decreased (CCC_20 = 18.27 g - CCC_3 = 15.54 g) with respect to the whole CCC (CCC_100 = 23.04 g) (**Table 3**). However, the NTP (CCC_20 = 11.59 tubers - CCC_3 = 14.99 tubers; **Figure 6C** and **Table 3**) and TTY (CCC_20 = 0.73 kg/plant - CCC_3 = 0.92 kg/plant; **Figure 6D** and **Table 3**) traits presented a similar tendency in mean values as Ho; here, the mean values in each of the core collections were greater than in the whole CCC (in CCC_100: NTP = 11.64 tubers and TTY = 0.77 kg/plant).

**Figure 7:**
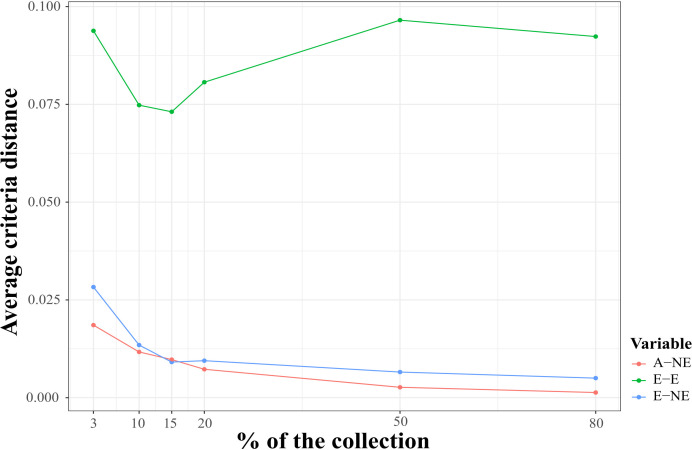
Evaluation of core quality using three different distance-based metrics for core identification and phenotypic data. The average criteria distance (y-axes) for three distance-based metrics A-NE (average Accession to Nearest Entry distance), E-E (average Entry to Entry distance), and E-NE (average Entry to Neatest Entry distance) calculated using phenotypic distance among core entries identified with A-NE criterion and genomic markers for six core collection sizes: 3, 10, 15, 20, 50 and 80 percent of whole samples genotyped in this study (x-axes).

Moreover, we compared the behavior of three distance-based metrics used for core collection identification to validate ― using phenotypic data ― the quality of the core and mini-core collections identified with SNP markers and the A-NE metric (**Figure 7**). As observed for the genotypic data, small values were obtained for the average A-NE distance for the different core collection sizes. Even though the average distances for E-NE and EE metrics were greater than A-NE, they did not show a strong maximization of values for small collection sizes. As expected, the average A-NE values decreased in the same way that the core size was closer to the full collection size.

### Core collection description

The 10 percent core and 3 percent mini-core collections contain accessions from different taxonomic groups and geographic origins (**Supplementary Table S1**). Of the 129 core collection accessions contained in the 10 percent collection, 82 accessions are *S. tuberosum* subsp. *andigenum*, 18 are *S. phureja*, 17 are *S. tuberosum* subsp. *tuberosum*, and 12 are currently undergoing curation. This 10 percent core collection contains 70 accessions from ten collection sites in Colombia, the larger representation from Nariño (22), Boyacá (14), and Cundinamarca (14) departments, followed by Cauca (6), Antioquia (5), Valle del Cauca (5), and one accession from Caldas, Quindío, Santander, and Tolima. Moreover, the core collection has accessions from Peru (7), Bolivia (3), Ecuador (2), Mexico (2), the USA (2), the Netherlands (2), and Germany (2). Thirty-nine of these accessions are of unknown origin. Of the 39 accessions in the mini-core collection (3 percent), 25 are *S. tuberosum* subsp. a*ndigenum*, six are *S. phureja*, two are *S. tuberosum* subsp. *tuberosum*, and six have yet to be identified and are in the process of curation. Regarding their origins, the mini-core collection has 22 accessions from Colombia collected from seven departments: Nariño, Cundinamarca, Boyacá, Antioquia, Cauca, Quindío, and Valle del Cauca. The mini-core collection has four accessions from other countries, two from Peru, one from Mexico, and one from the Netherlands. Finally, the mini-core collection has 13 accessions of unknown origin.

## Discussion

Our study genotyped 90.9 percent (1141/1255) of the clonal collection of the CCC of potatoes and a representation of released varieties. These genomic data allowed us to study the population structure and genetic diversity, determine ploidy, and define core and mini-core collections representing the genetic diversity of the whole CCC. Below we discuss our study results and how we can use them to curate and improve the Colombian potato collection in agreement with the Global Conservation Strategy for potato (Nagel et al., 2022).

### Population structure and genetic diversity

The pattern of population genetic structure and diversity observed suggests that potato accessions held at the CCC fit the evolutionary patterns of farming selection that separated diploid from tetraploid landraces, and this from the most-modern cultivated potato (*Solanum tuberosum* L.). According to our previous study that used 809 accessions (Berdugo-Cely et al., 2017), the CCC has a population structure that contains two main genetic groups of mostly diploid and tetraploid accessions represented in general by the Phureja and Andigenum types, respectively (CCC_Group_A and CCC_Group_B). This study corroborated this pattern, but separated the tetraploid genetic group into two well-differentiated genetic groups (CCC_Group_B1 and CCC_Group_B2). The CCC_Group_B1 has a greater number of accessions and levels of admixture than the two other genetic groups. Most accessions are of Andigenum type, probably with more primitive ancestry than CCC_Group_B2. Thus, the Andigenum accessions that resulted in a more differentiated and divergent CCC_Group_B2 genetic group probably come from a more recent selection pressure by farmers and breeders for some modern traits. A higher number of Tuberosum accessions were also in CCC_Group_B2 group, supporting the hypothesis that this group suffered substantial selection pressure. This concurs with the fact that most of the Tuberosum accessions in the CCC are modern varieties acquired as part of potato breeding efforts in Colombia (Moreno and Valbuena, 2006). Differences in the agricultural selection patterns have been reported in potatoes. The modern North American and traditional Andean cultivars shared only 14−16 percent of genes under selection; thus, the adaptation of highland Andigenum and lowland Tuberosum had different selection strategies (Hardigan et al., 2017). Besides this conclusion, we consider that modern breeding practices in North America and Europe also strengthened the differentiation between most modern/commercialized potato and Andean landraces. The CCC_Group_B1 and CCC_Group_B2 groups also showed divergence from Phureja, probably due to polyploidization, a more comprehensive distribution range, and the selection pressure for some culinary or crop traits. In general, potatoes have been selected by ancient farmers and, more recently, by breeders for tuber size, tuber carbohydrates and glycoalkaloids content, photoperiod adaptation, and reduced sexual fertility (Hardigan et al., 2017). The CCC diversity study results support divergence between diploid and tetraploid accessions from Phureja, Andigenum, and Tuberosum types, probably mediated by selection patterns.

The CCC is one of the Latin American clonal native potato collections that can be compared to the International Potato Center (CIP) collection in Peru, with around 4,500 landrace accessions (Ellis et al., 2020). Genetic diversity analysis of part of CIP’s potato collection agreed with our analysis results for the patterns of genetic differentiation of the Phureja and Andigenum groups observed in the CCC. Thus, Andigenum has a more significant number of accessions, genetic admixture, and genetic diversity. In contrast, Phureja is more genetically homogeneous with fewer accessions (Ellis et al., 2018). As documented by different authors, the genetic diversity of native tetraploid potatoes from the Andigenum group has broader geographic coverage and diversity compared to the Phureja group distributed in a more restricted area (Hawkes, 1990; Huamán and Spooner, 2002; Spooner et al., 2010; Bradshaw, 2021), which suggests that polyploidy gave advantages of yield, robustness, and adaptation to different environments in the Andean Highlands. Although the Phureja diploids are also derived from *S. stenotomum*, they were selected due to a lack of tuber dormancy, shorter crop cycles, and adaptability to warmer, lower, and eastern valleys of the Andes. Besides the less cosmopolitan adoption of diploid Phureja, some levels of self-compatibility could also have contributed to the genetic homogeneity of this group. While the self-incompatibility nature of diploid potatoes is widely acknowledged, recent reports have revealed the existence of self-compatibility sources in cultivated potatoes (Kaiser et al., 2021). Notably, the Phureja clone 1S1 has been identified as a source of self-compatibility, leading us to the assumption that self-compatibility contributes to the genetic diversity of Phureja (ibid). In summary, both the CIP and CCC potato collections exhibited similar patterns of genetic diversity differentiation for Phureja and Andigenum accessions.

In different population structure and genetic diversity studies of genebank collections of traditional cultivars and breeding programs germplasm of potato, the material has been discriminated following ploidy, taxonomy, and characteristics of breeding selection (Hirsch et al., 2013; Berdugo-Cely et al., 2017; Hardigan et al., 2017; Deperi et al., 2018; Ellis et al., 2018; Pandey et al., 2021). In general, it is observed that the genetic diversity studies of germplasm obtained from genebanks have shown that the discrimination among accessions is mainly associated with ploidy and taxonomy (Berdugo-Cely et al., 2017; Hardigan et al., 2017; Ellis et al., 2018; Berdugo-Cely et al., 2021). This discrimination agrees with our results, in which ploidy and taxonomy explained the identified genetic groups. In contrast, genetic diversity studies of germplasm from breeding programs have shown that discrimination of accessions depends on potato market class selection, indicating that the diversity is shaped by the strong selection pressure for breeding traits (Hirsch et al., 2013; Deperi et al., 2018; Pandey et al., 2021). Overall, the studies on population structure and genetic diversity in potato showed that trait introgression from wild germplasm and the selection pressure of breeding contributed to increasing heterozygosity and diversification of cultivated lineages.

### Ploidy assessment

This study used the frequency of simplex and triplex scores of all SNPs in each accession as a ploidy level predictor, following Alsahlany et al. (2019). This methodology is possible because the SNP genotyping using the Illumina array technology allows the dosage of biallelic markers in an autotetraploid species such as a potato to be identified. Alsahlany et al. (2019) proposed assessing the ploidy based on the frequency of simplex and triplex SNP scores (ABBB, AAAB), in which one allele would be present in a higher proportion than the other. This approach is objective because diploid accessions would only have two alleles, giving the same proportion for each; thus, there is a low or null probability of detecting a simplex or triplex SNP score in a diploid. Paralogous genes or mismatches between the sample DNA and the SNP probe could explain the detection of simplex or triplex scores in diploids, but this has a low probability of occurring.

In this study, the frequency of simplex and triplex SNP scores identified in the reference set of samples varied compared to Alsahlany et al. (2019); tetraploids have a proportion of 27.1 percent ±1.53 compared to 36 percent ±14, and diploids 4.8 percent ±9.04 compared to 2 percent ±1. The separation threshold to predict our samples’ ploidy was <15 percent for diploids and >20 percent for tetraploids. Accessions with frequencies between 15 and 20 percent were considered unknown; this classification was tight compared with the ample separation reported by Alsahlany et al. (2019). The simplex/triplex frequency was up to 4 percent for most diploid samples (263/297, 88.6 percent), and a few samples were 9−15 percent (34/297, 11.4 percent). The genetic diversity of this collection probably interfered with the DNA hybridization during SNP genotyping for those samples and affected the proportion of simplex and triplex SNP genotype scores. In contrast, Alsahlany et al. (2019) used North American breeding germplasm similar to the varieties used to develop the potato SNP array (Hamilton et al., 2011). Even though for a few samples, the parameters for classification used to assess the ploidy of accessions in the CCC based on the frequency of simplex and triplex SNP scores were tight, this approach helped estimate the ploidy in the CCC.

Heterozygosity values estimated for potato samples genotyped with the Infinium Potato Array have also been related to ploidy levels. Ellis et al. (2018) reported that each ploidy group of a panel of 500 samples of potato landraces was associated with a percentage of heterozygosity pattern. However, they also detected that heterozygosity overlaps in narrow ranges making the separation difficult. They found higher heterozygosity in tetraploid germplasm (>30 percent) compared to diploids (<20 percent), and triploids have 20−30 percent of heterozygosity. In the present study, we found that the ranges of heterozygosity for samples in the reference set, excluding potential errors, were 10−23 percent for diploids, 13 percent for triploids, and 33−58 percent for tetraploids. However, there was a continuous data distribution of 9−66 percent heterozygosity for the complete samples with no clear separation. We expected mainly diploids, tetraploids, and a low number of triploids in the CCC (**Figures 2C**, **3C**). These results showed that the separation thresholds should be defined for each case based on the germplasm evaluated. Similarly, the frequency of simplex and triplex scores of all SNPs in one accession is a much better approach to predicting ploidy level than the heterozygosity values.

A reference set of samples with ploidy information generated by other indirect (chloroplast count per guard cell or flow cytometry) and/or direct (chromosome count) methods would better define the threshold separation in ploidy assessment. In our case, we compared data from SNP scores, the number of chloroplast/guard cells, and chromosome counts. The threshold separation for the number of chloroplast/guard cells (6−8 for diploids and 12−14 for tetraploids) was consistent with previous reports (Rasmussen and Rasmussen, 1995; Gebhardt et al., 2006; Ordoñez et al., 2014; Alsahlany et al., 2019). Although assessing the ploidy using only SNP scores is valid, we recommend confirming this assessment with a low-cost and time-efficient technique such as the chloroplast count in guard cells.

### Core collection

We proposed the 10 percent subset as the core collection because it already captured the representation of the total diversity of the CCC. The reduced but highly diverse core collection has a more manageable and versatile number of accessions for trait discovery, evaluations, and use in Colombia’s potato breeding and crop improvement. The representation of phenotypic diversity for three agronomic traits (ATW, NTP, and TTY) within the core and mini-core collections confirmed their potential use. In general, the evaluation of the CCC for sources of new alleles for different traits has taken several years because of the size of this collection. Now, faster screening can be conducted, and the mini-core collection, which is even smaller in size, represents an alternative when evaluating the core collection is not feasible.

As expected, after eliminating redundancy in the collection, the genetic diversity measured using the Ho average increased in the core and mini-core collections. Similar results have been reported for recently developed core collections of potato (Bamberg and del Rio, 2004; Pandey et al., 2021). The PCA analysis also showed that the sample dispersion in the scatter plots has a similar distribution across core collection sizes. Likewise, our behavior analysis of the average distance-based metrics A-NE, EE, and E-NE used to identify core and mini-core collections confirmed that the A-NE metric is robust for obtaining a uniform representation of the original genetic space, as proposed by Odong et al. (2013). A-NE algorithm aims to obtain a small average distance between accession and the nearest entry in the core for the whole collection; this guarantees the representativity of all accessions in the core collection. By contrast, the E-E and E-NE methods maximize the average genetic distances between entries and entry to nearest entry to identify core collections representing extreme values or a distribution, respectively. The average A-NE also decreased as the collection size increased because, as expected, the maximum representation of each accession by an entry is itself. The identified core and mini-core collections followed the expected average A-NE performance using phenotypic data.

The selected 10 percent core collection size was within the range of 5−20 percent core collection size reported and corresponded with the most recommended size (Van Hintum et al., 2000). We will present this core collection to breeders and scientists in AGROSAVIA to obtain their feedback and contributions to ensure the selected entries are the most suitable for research and breeding. If needed, the accessions of research and breeding interest or with long historical evaluation records could be added to the core collection by replacing initially selected entries with those that have the most similar genetic distance.

### Practical use of diversity and ploidy assessment in the management of CCC

The CCC has been curated with the support of diversity studies and ploidy assessment. The clonal CCC of potatoes has been under AGROSAVIA´s management since 1994. At that time, the collection handled by the former curator categorized the accessions into different groups: Phureja, Andigena, Tuberosum, “Guata,” “Chaucha,” and unknown. This classification is based on a combination of morphological patterns specific to Andigenum, Phureja, and Tuberosum taxonomic classification, and a non-taxonomic, ethnobotanical classification for certain native accessions or breeding germplasm, referred to as “Guata,” and “Chaucha”. This AGROSAVIA categorization has been adjusted for each accession during the CCC curation process to assign and/or verify the classification according to the taxonomic classification of Hawkes (1990). Initially, the collection was morphologically characterized to document the discriminatory descriptors in the CCC and the characteristics of each accession. Then, the digitalization of passport data with the originally assigned taxonomic classification information, genetic diversity analysis, and ploidy assessment supported curation of the classification. In previous years, morphological descriptors and genetic diversity analysis were used to reassign some Chaucha accessions into the Phureja group. For the accessions evaluated in this study, we found that the Guata classification corresponds to accessions that are mostly tetraploids (83/88, 94,3 percent) and, based on a few recovered passport data (23/88), the accessions also correspond to *S. tuberosum* subsp. *andigenum*. The assignment to genetic groups, ploidy, morphology, and historical records should be considered when reassigning taxonomic classification. Some diploid accessions can be Andigenum, and some tetraploid accessions Phureja. In potatoes, the germplasm ploidy can be increased due to crosses involving 2n gametes (polyploidization) or reduced by pickle pollination (haploidization). Phureja accessions are mainly diploids, but triploids and tetraploids can be found in a low proportion (Ghislain et al., 2006). Similarly, Andigena accessions are mainly tetraploids; however, di-haploids could have been generated as part of breeding program strategies. Thus, the morphological description, passport data, analysis of diversity, and ploidy assessment need to be continued to support the curation of this collection.

In Colombia and Ecuador, indigenous communities and traditional farmers categorize the native cultivars between Chauchas and Guatas. In general, cultivars with a short crop cycle and early sprouting potato are known as Chauchas, which means *soft or easy*, and cultivars with an annual crop cycle and white tuber flesh color as Guatas, a Quechua word meaning *potato of one year* (Monteros-Altamirano, 2017; Rosero Alpala et al., 2020). From a taxonomic perspective, the indigenous or ethnobotanical classification of Chaucha contains germplasm that mainly corresponds to the formerly accepted classification by Hawkes (1990) of *S. phureja* and in lesser proportion to *S. chaucha*, while the Guatas are mainly associated with the *S. tuberosum* subsp. *andigenum* (Navarro et al., 2010; Monteros-Altamirano, 2017; Rosero Alpala et al., 2020). This homologation using ethnobotanical and taxonomic classification is fully aligned with the results obtained in the curation of the CCC collection (**Figure 1A**), thus this type of information could support taxonomic classification.

Part of the curation process is keeping all the information related to the germplasm organized by attributes or categories. The database should contain information on common names, taxonomic classification based on different authors (Hawkes, Spooner), biological status (wild, native/landrace, and breeding\research germplasm), and ethnobotanical classification (Chauchas and Guatas). Regarding the different taxonomic classifications, it is important to maintain the information of Hawkes’ classification, which facilitates the management and use of germplasm in the conservation and breeding programs. Even though the classification based on Spooner et al. (2007) that regroups four former species (*S. phureja*, *S. tuberosum* subsp. *andigenum*, *S. stenotomum*, and *S. chaucha*) into a single species is well supported, a suite of morphological descriptors, length of plant cycle, photoperiod adaptation, and cytoplasm type support their differentiation (Hawkes, 1990; Spooner et al., 2014). If this distinction is unclear, scientists and breeders can encounter issues with fertility, dormancy, tuber attributes, tuberization, and maturity traits.

### Future perspectives for CCC management

This study successfully genotyped 90.9 percent of the clonal collection of the CCC of potatoes and a representation of released varieties. This result is an outstanding achievement considering this collection’s size (2,499 wild and cultivated potato accessions) and its importance for the region. Moreover, we are interested that this collection become in a regional reference. Therefore, we would recommend aligning the CCC conservation strategy with the ten actions suggested for improving the global-scale conservation of potato resources, as proposed by several institutions and organizations (Nagel et al., 2022). Some of the actions that can be taken after this study include finding duplicates, clarifying the possible accession admixture exposed in this study, determining the ploidy using the chloroplast count in guard cells, verifying the taxonomy and biological status for accessions in the curation process, completing the digitalization of documentation and centralization of all historical and new evaluation data in the GRIN-Global platform, prioritize future evaluations in field experiments and data collection using the core collection, and completing the evaluation of core accessions that have missing information from previous biotic and abiotic evaluations. This collection’s passport data, morphological characterization, and genetic profiling can be used to compare the *in vitro* and field collection to verify the identity of germplasm. Ellis et al. (2018) showed that some accession admixing could happen over time in the genebanks. Therefore, through an identity verification process, the collection can be revised, documented, and organized to correct errors and mitigate future mistakes by digitalizing data and implementing barcoding and quality management standards developed for genebanks.

The genotyping data generated in this study can also be compared with other collections, such as CIP’s collection, genotyped using the same technology. This comparison could serve to identify signatures of selection, materials with potential use in breeding, duplicity, genetic gaps, homologate accessions between the collections, and assess the extent to which CCC diversity is representative of the world’s largest collection of native potatoes. This paper is also available in Spanish to continue the discussion of this topic within Colombia and more broadly in the region (**Supplementary Data Sheet 1**).

## Data availability statement

The datasets presented in this study can be found in online repositories. The names of the repository/repositories and accession number(s) can be found in the article/**Supplementary Material**. 

## Author contributions

RY, JB-C, IC-S, and PR-H conceived the study. JB-C, ZL-P, and PR-H organized the CCC databases and designed the analysis. ZL-P and IC-S collected leaves from selected accessions. JB-C and IC-S worked in the wet lab. JB-C and PR-H performed the analysis, including descriptive graphics. NM-C led the manuscript writing and performed part of the analyses. All authors contributed to the article and approved the submitted version. 

## Supplementary material

Data Sheet 1 Contains translated material.

Supplementary Figure 1 Number of genetic clusters suggested by **(A)** STRUCTURE Harvester **(B)** and NBClust R-Package test for 1,291 genotyped samples of the Colombian Central Collection (CCC) of potatoes and breeding material using 3,586 Single Nucleotide Polymorphism (SNP) markers.

Supplementary Table 1 List of potato genotype accessions of the Colombian Central Collection (CCC) and breeding material analyzed in this study.

Supplementary Table 2 Genotype matrix that included the 3,586 genome-wide single nucleotide polymorphic (SNP) markers identified and filtered for 1,291 potato samples of the Colombian Central Collection (CCC) and breeding material analyzed in this study.
