# Supplementary material for: Defining a diverse core collection of the Colombian Central Collection of potatoes: a tool to advance research and breeding
Source: Front Plant Sci. 2023 Apr 26;14:1046400. doi: 10.3389/fpls.2023.1046400 (PMC10173156; doi:10.3389/fpls.2023.1046400)
Supplement: Data Sheet 1 — Contains translated material. [file DataSheet_1.docx]

**Establecimiento de una colección núcleo con la diversidad de la Colección Central Colombiana de papa: Una herramienta para avanzar la investigación y el fitomejoramiento**

Manrique-Carpintero, N. C.^1,2^, Berdugo-Cely, J. A.^1,3^, Cerón-Souza, I.1, Lasso-Paredes, Z.^1^, Reyes-Herrera, P^1^, Yockteng R.^1,4^**^*^**

^1^ Corporación Colombiana de Investigación Agropecuaria-AGROSAVIA. Centro de Investigación Tibaitatá. Km 14 vía Mosquera-Bogotá. Mosquera. Cundinamarca. Colombia.

^2^ Alianza de Bioversity Internacional y el Centro Internacional de Agricultura Tropical (CIAT). Km 17 vía Cali-Palmira. Cali. Colombia

^3^Corporación Colombiana de Investigación Agropecuaria-AGROSAVIA. Centro de Investigación Turipaná. Km 13 vía Montería-Cereté. Montería. Córdoba. Colombia.

^4^Institut de Systématique, Evolution, Biodiversité-UMR-CNRS 7205. National Museum of Natural History. France.

***Correspondencia**

**Autor de correspondencia**: [ryockteng@agrosavia.co](mailto:ryockteng@agrosavia.co)

**Resumen**

La gran diversidad de la Colección Central Colombiana (CCC) de papa cultivada, es la fuente más importante de variación genética para el fitomejoramiento y el desarrollo agrícola de este cultivo básico en Colombia. La papa es la principal fuente de ingresos para más de 100.000 familias campesinas en Colombia. Sin embargo, diferentes desafíos bióticos y abióticos limitan la producción del cultivo. Además, nuevos retos debido al cambio climático, la malnutrición y la necesidad de garantizar seguridad alimentaria exigen que se aborde urgentemente el desarrollo de cultivos resilientes. La CCC clonal de papa contiene 1.255 accesiones, un tamaño de colección bastante grande que limita su óptima evaluación y uso. En nuestro estudio se evaluaron diferentes tamaños de colección a partir de la colección principal con el fin de identificar la mejor colección núcleo que representará toda la diversidad genética de esta colección única. La colección núcleo permitirá hacer una caracterización más efectiva y rentable de la diversidad en la CCC. Inicialmente, se genotiparon 1.141 accesiones de la colección clonal y 20 líneas de mejoramiento utilizando 3.586 marcadores polimórficos a nivel genómico para estudiar la diversidad genética de la CCC. El análisis de varianza molecular confirmó que la CCC es diversa y que estadísticamente la población tiene estructura genética (Phi=0,359; p-valor=0,001). Se identificaron tres grupos genéticos principales dentro de esta colección (CCC_Group_A, CCC_Group_B1, y CCC_Group_B2), y las variedades comerciales se localizaron a través de estos grupos. El nivel de ploidía fue el principal factor determinante de la identificación de los grupos, seguido de una sólida representación de accesiones con clasificación taxonómica de los grupos de cultivares Phureja y Andigenum según la clasificación previamente aceptada. También encontramos valores de heterocigosidad divergentes dentro de los grupos genéticos, con mayor diversidad en los grupos genéticos con accesiones tetraploides (CCC_Group_B1: 0,37, y CCC_Group_B2: 0,53) que en los de diploides (CCC_Group_A: 0,14). Posteriormente, generamos una colección mini-núcleo de un 3% (39 entradas) y tres colecciones núcleo de un 10%, 15% y 20% del total de muestras genotipadas (conteniendo 129, 194 y 258 entradas, respectivamente). Como nuestros resultados mostraron que la diversidad genética en los diferentes tamaños de las colecciones núcleo que se muestrearon era similar al de la colección principal, seleccionamos el tamaño de colección núcleo más pequeño, es decir, el 10%. Esperamos que esta colección núcleo del 10% sea una herramienta óptima para descubrir y evaluar la diversidad funcional en el banco de germoplasma, con el fin de que contribuya en el avance del mejoramiento genético de la papa y en los estudios relacionados con la agricultura. Este estudio también sienta las bases para continuar la curación de la CCC con la evaluación de duplicados y mezclas entre accesiones, la finalización de la digitalización de los datos y la determinación de la ploidía mediante el método de conteo de cloroplastos.

**Palabras clave:** diversidad genética, marcadores moleculares; estructura de la población; colección mini-núcleo, poliploidía.

**INTRODUCCIÓN**

La diversidad de los cultivos es la principal fuente de variación genética para el mejoramiento genético de los cultivos y el desarrollo de las especies cultivadas. La papa (*Solanum tuberosum* L.) es una especie con una gran diversidad en el acervo genético primario. A medida que tuvo lugar la domesticación, co-evolucionaron varias especies cultivadas, desde la papa cultivada más ancestral, *S. stenotomum*, hasta la papa más moderna y comercializada globalmente (Spooner et al., 2014). Según la clasificación taxonómica más reciente, existen cuatro especies cultivadas de papa, *Solanum tuberosum* L., con dos grupos de cultivares, Andigenum y Chilotanum, *S. ajanhuiri*, *S. juzepczukii* y *S. curtilobum* (Spooner et al., 2007). En el grupo Andigenum fueron fusionadas varias especies de la clasificación taxonómica de Hawkes (1990), que era la clasificación previamente aceptada. Se unió en una especie *S. stenotomum* Juz. & Bukasov, *S. phureja* Juz. & Bukasov, *S. chaucha* Juz. & Bukasov, y *S. tuberosum* subsp. andigenum Hawkes. Diversos factores dinámicos y evolutivos asociados al cultivo tradicional de la papa por las comunidades Andinas generaron la amplia diversidad genética de las papas cultivadas. Dichos factores son la hibridación continua de la diversidad cultivada promovida por la naturaleza alógama de la mayoría de los diploides, la producción de gametos no reducidos, la presencia de diversidad cultivada con diferentes niveles de ploidía en un mismo campo, la reproducción sexual y asexual de forma simultánea dando prioridad a la propagación vegetativa, y los posibles cruces con germoplasma silvestre que crece cerca de los campos cultivados. Todos estos factores promovieron la generación y el mantenimiento a lo largo del tiempo de la enorme variación de las variedades de papa cultivada (Dodds y Paxman, 1962; Huamán y Spooner, 2002; Spooner et al., 2010).

Las variedades tradicionales/nativas/criollas/locales de papa son originarias de América del Sur; la mayoría de ellas provienen de las tierras altas de los Andes (entre 3000 y 4000 m de altitud), desde el oeste de Venezuela hasta el sur, en el sur de Bolivia y el norte de Argentina, a excepción de las variedades tradicionales del grupo Chilotanum que se encuentran en las tierras bajas de la zona central y centro-sur de Chile (Spooner et al., 2010). Las variedades cultivadas en Centroamérica y México proceden de introducciones postcolombinas. Con base en el análisis de la distribución ecogeográfica de las variedades locales de papa, el grupo *S. tuberosum* Andigenum tiene una cobertura geográfica más amplia, siendo los tetraploides los que tienen una mayor distribución, seguidos por los niveles de ploidía diploide y triploide con menor extensión. Al igual que el grupo Chilotanum, *S. ajanhuiri*, *S. curtilobum* y *S. juzepczukii* tienen una distribución mucho menor, restringida al centro de Perú y Bolivia. De esta amplia distribución, se han reportado varios miles de variedades nativas de papa con una amplia diversidad genética que puede ser descrita en varios niveles: entre los que el morfológico, fisiológico y agronómico, son algunos a destacar. Diferentes autores han reportado una amplia diversidad en el color de la piel y la pulpa del tubérculo, la forma del tubérculo y de la hoja, los colores florales, el hábito de crecimiento, la madurez, la dormancia y la duración del fotoperiodo necesario para la tuberización, además de la resistencia a plagas y enfermedades, la capacidad de respuesta ambiental y el rendimiento del tubérculo (Spooner et al., 2010; Ellis et al., 2020; Bradshaw, 2021). Esta amplia diversidad representa una reserva de genes importantes para el mejoramiento de los cultivos, la seguridad alimentaria y la adaptabilidad al cambio climático (ibid).

Colombia se considera centro de diversidad de la papa, ya que es uno de los países Andinos donde se domesticó el cultivo. La papa es un importante alimento básico para la seguridad alimentaria en Colombia y la principal fuente de ingresos para alrededor de 100.000 familias campesinas (Ministerio de Agricultura y Desarrollo Rural, 2020). La diversidad de la papa en Colombia se conserva *in situ* y *ex situ*. La conservación *in situ* es realizada por algunas comunidades indígenas aisladas y por pequeños agricultores que aún conservan variedades tradicionales (Tinjacá y Rodríguez, 2015). Esto se debe a que el cultivo de papa en la mayor parte del territorio utiliza prácticas comerciales modernas. Por otro lado, la conservación *ex situ* de la papa es realizada por la Corporación Colombiana de Investigación Agropecuaria (AGROSAVIA) y universidades como la Universidad Nacional de Colombia, entre otras. Se utilizan tres sistemas diferentes: semillas, campo e *in vitro*. Los agricultores y las empresas privadas también intentan conservar y promover la comercialización de las papas nativas. La Colección Central Colombiana (CCC) de papa se inició en la década de 1950 como base de una plataforma genética para la iniciativa de investigación y mejoramiento de papa promovida por varios científicos de plantas, agrónomos y agricultores colombianos, apoyada por el Ministerio de Agricultura de Colombia y guiada por el Dr. Jack Hawkes (Luján, 1976; Pineda Colorado y Hernández Castillo, 1996) quien estableció el programa nacional de mejoramiento de papa. En la actualidad, AGROSAVIA administra la CCC y conserva 2.499 accesiones de papa silvestre y cultivada. La colección clonal de germoplasma cultivado cuenta con 1.255 accesiones, la mayoría de las cuales pertenecen al Grupo *S. tuberosum* Andigenum y unas pocas accesiones al Grupo *S. tuberosum* Chilotanum según Spooner et al. (2014). Sin embargo, en la CCC el germoplasma se clasifica como *S. phureja*, *S. tuberosum* subsp. andigenum y *S. tuberosum* subsp. *tuberosum*, siguiendo la clasificación taxonómica de Hawkes (1990).

La diversidad de la CCC ha sido importante para avanzar la investigación y el mejoramiento del cultivo de papa en Colombia. Específicamente, la evaluación de parte de la CCC clonal mostró que es altamente diversa, con una estructura poblacional alta y significativa y con mayor variación genética dentro de las poblaciones que entre ellas (Juyó et al., 2015; Berdugo-Cely et al., 2017). Esta colección ha sido usada como fuente de resistencia al tizón tardío causado por *Phytophthora infestans*, a la polilla guatemalteca del tubérculo *Tecia solanivora* y al estrés por sequía (Santa Sepúlveda, 2018; Díaz-Valencia et al., 2021). Se considera que estas son algunas de las limitantes más importantes para la producción del cultivo de papa. Además, la CCC ha sido usada para generar y liberar nuevas variedades en los últimos 70 años en Colombia (Pineda Colorado y Hernández Castillo, 1996; Ñustez, 2011). De igual forma, en 1995 científicos de AGROSAVIA iniciaron la caracterización morfológica de la CCC, junto con diferentes evaluaciones de caracteres esenciales para el mejoramiento tales como resistencia a tizón tardío, madurez y atributos de calidad (Moreno y Valbuena, 2006; Berdugo-Cely et al., 2017). La mayoría de las evaluaciones fueron realizadas por grupos de accesiones por lo que tomo un largo período de tiempo, esto debido al gran tamaño de la colección y a los limitados recursos a diferentes niveles, como personal capacitado, financiación, infraestructura, etc. El cultivo de papa en Colombia está sujeto a varios retos bióticos y abióticos, además de las problemáticas de seguridad alimentaria, desnutrición y cambio climático. Por ello, la CCC ha sido y seguirá siendo la principal fuente de diversidad genética para el mejoramiento del cultivo en Colombia.

El tamaño de una colección, en particular de las colecciones grandes, suele limitar la posibilidad de documentar y utilizar plenamente el germoplasma debido a la elevada cantidad de recursos logísticos y de infraestructura requeridos para llevar a cabo la investigación y la evaluación de la colección. Por ello, Frankel (1984) propuso racionalizar las colecciones, reduciendo la redundancia y generando colecciones núcleo, un subconjunto representativo de toda la diversidad de la colección. En general, una colección núcleo pretende maximizar la representación de toda la diversidad genética. Sin embargo, una colección núcleo también puede ensamblarse para capturar la diversidad fenotípica de rasgos contrastantes o extremos, o para representar patrones de diversidad basados en diferentes criterios como la distribución geográfica, la proporción de diversidad por conglomerados y la diversidad de un rasgo, entre otros (Odong et al., 2013). En una colección núcleo -diseñada para representar toda la diversidad de la colección principal- cada accesión o entrada seleccionada representa a todas las accesiones de la colección principal que son similares a ella. Por lo tanto, cada entrada de la colección núcleo, podría representar una o más accesiones de la colección completa. Esto es posible porque la selección se produce a partir del centro de conglomerados únicos o grupos que conforman una representación uniforme en toda la diversidad. Odong et al. (2013) recomendaron utilizar métricas basadas en la distancia genética para seleccionar las entradas de una colección núcleo que representen la diversidad genética, exactamente el promedio mínimo de la distancia entre cada accesión y la entrada más cercana (A-NE por la sigla en inglés *Average Accession to Nearest Entry*). Por lo que, para garantizar una buena selección de una colección núcleo, el valor de la distancia A-NE debería ser el menor posible, considerando que en sí misma (A-NE=0) es la máxima representación de una accesión. Dado que una métrica basada en la distancia genética es el mejor criterio para generar una colección núcleo de diversidad, deben utilizarse marcadores moleculares en lugar de datos fenotípicos o de pasaporte, para encontrar una colección núcleo que maximice la representación de toda la diversidad genética contenida en la colección principal. La mayoría de los marcadores moleculares son selectivamente neutros, no se ven afectados por el medio ambiente y son más adecuados para el análisis estadístico de la diversidad genética (Brown, 1989; Chapman y Crawford, 1990). Desarrollar muchos marcadores con cobertura a nivel genómico, que garanticen la mejor representación de la diversidad genética por las entradas seleccionadas es relativamente viable hoy en día.

En este estudio se evaluó la estructura de la población y la diversidad genética de la CCC utilizando polimorfismos de nucleótido simple (SNP por la sigla en inglés *Single Nucleoide Polymorphism*) de todo el genoma. Del mismo modo, se crearon colecciones núcleo y mini-núcleo utilizando el método de promedio de la distancia entre cada accesión y la entrada más cercana (A-NE por la sigla en inglés) y la validación recomendada por Odong et al. (2013). La mejor colección núcleo y mini-núcleo se identificaron comparando la medida de diversidad genética de cada una con la de toda la CCC. Posteriormente, la calidad y utilidad de la colección núcleo seleccionada se validó comparando la representación de la diversidad fenotípica propia de la CCC con de las colecciones núcleo y mini-núcleo para tres rasgos agronómicos diferentes (Peso Promedio del Tubérculo en g (PPT), Número de Tubérculos por Planta (NTP), y Rendimiento Total del Tubérculo (RTT) en kg/planta). Por último, con este estudio sentamos las bases para continuar la curación de la CCC evaluando duplicados y mezclas entre accesiones, completando la caracterización, la digitalización de datos y la determinación de la ploidía mediante el método de conteo de cloroplastos.

**MATERIALES Y MÉTODOS**

**Material vegetal**

Para este estudio se utilizaron 1.141 accesiones de la CCC de papa mantenidas en campo o *in vitro* y que forman parte del banco de germoplasma administrado por AGROSAVIA y 20 variedades comerciales y líneas de mejoramiento avanzadas obtenidas de diferentes fuentes. La CCC contiene principalmente variedades nativas y una pequeña proporción de variedades liberadas. Basándose en la taxonomía de Spooner et al. (2007), la mayoría de las accesiones pertenecen a la especie *S. tuberosum* Grupo Andigenum, y pocas accesiones a *S. tuberosum* Grupo Chilotanum. Sin embargo, la clasificación del material de la colección de papa sigue la clasificación taxonómica previamente aceptada de Hawkes (1990). De acuerdo con esta clasificación, 143 accesiones son *S. phureja*, 681 *S. tuberosum* subsp. *andigenum*, 67 *S. tuberosum* subsp. *tuberosum*, una (1) *S. rybinii* (clasificación más antigua), mientras que para 269 accesiones actualmente no se cuenta con esta información, debido a que se encuentra en proceso de curación y digitalización de los datos de los registros impresos. Basándose en la clasificación original, este estudio ha nombrado las accesiones como tipos Andigena, Phureja y Tuberosum. El origen geográfico de las accesiones es diverso, se tienen registros de 802 accesiones, de las cuales 670 son de Colombia, 95 de otros países de Latinoamérica, 26 de Europa y 11 de Estados Unidos (Tabla 1).

**Muestreo y genotipado**

Para cada accesión se recolectaron muestras de tejido foliar de plántulas *in vitro* de 1-2 meses de edad o de plantas cultivadas en campo en estado vegetativo. En el segundo caso, el tejido se recogió de una planta seleccionada al azar de uno de los 20 clones cultivados por accesión. Los curadores de papa de la CCC regeneran anualmente la colección de campo en una zona montañosa del municipio de Zipaquirá, Cundinamarca, Colombia, a 2.950 m de altitud, con una temperatura promedio de 15°C y una humedad relativa de 75%. Se extrajo el ADN del tejido foliar utilizando el DNeasy Plant Mini Kit (Qiagen, Valencia, CA, USA), siguiendo las recomendaciones técnicas del fabricante. Luego, genotipamos cada accesión con el microarreglo de marcadores SNP de papa *Infinium* de Illumina versión 8303 (Felcher et al., 2012) y escaneamos las señales fluorescentes en el sistema Illumina HiScan SQ (Illumina, San Diego, CA) en AGROSAVIA. Un conjunto de 809 muestras ya tenía un perfil genético que fue procesado por Berdugo-Cely et al. (2017). Por lo tanto, se genotiparon 482 muestras adicionales para obtener un total de 1.291 muestras genotipadas y utilizadas en este estudio (Tabla Suplementaria S1). De estas muestras, 1.061 son accesiones únicas y 130 son repeticiones biológicas. Inicialmente, las accesiones de la CCC clonal no tenían números de identificación únicos, por lo que las muestras fueron colectadas de material en campo e *in vitro* utilizando otra nomenclatura. Durante el proceso de genotipado, y como parte del proceso de curación de la CCC, los números de identificación únicos fueron asignados a las accesiones; esto nos permitió identificar múltiples muestras que habían sido genotipadas varias veces. Debido a limitaciones presupuestarias, las muestras evaluadas en este estudio fueron genotipadas por lotes hasta completar todas las muestras, lo que contribuyó a acumular varios duplicados en el proceso de genotipado. Obtuvimos los valores brutos de las señales fluorescentes para todas las muestras utilizando el software GenomeStudio 2.0 (Illumina, San Diego, CA). El paquete Clustercall (Schmitz Carley et al., 2017) en la plataforma R (R Core Team, 2021) transformó estos valores de señal en códigos de genotipo tetraploide para cada marcador SNP (AAAA, AAAB, AABB, ABBB, BBBB codificadas como 0, 1, 2, 3, 4).

**Estructura de la población y diversidad genética**

La matriz final de genotipado con marcadores SNP se obtuvo a partir de la matriz de salida de ClusterCall, esta fue filtrada para eliminar los marcadores con frecuencia alélica mínima menor de 0,03 y con datos perdidos superiores al 5% tanto para genotipos como para marcadores. Evaluamos la estructura de la población utilizando este conjunto de datos con codificación tetraploide de genotipos y dos estrategias de análisis: i) un modelo Bayesiano implementado en el software STRUCTURE 2.3 (Pritchard et al., 2000) sin información *a priori* de la población, y ii) un Análisis de Componentes Principales (ACP) calculado utilizando el paquete R-Adegenet (Jombart y Ahmed, 2011). Para el modelo Bayesiano, el número de poblaciones (denominadas grupos genéticos en este estudio) K se estimó en un rango de uno a 10 utilizando un modelo de mezcla con frecuencias de alelos correlacionados, un *burn-in* fijado en 150.000 interacciones y diez repeticiones independientes por ejecución. Posteriormente, encontramos el mejor número de poblaciones con ayuda del programa Structure Harvester (Earl y vonHoldt, 2012) utilizando el método de Evanno (Evanno et al., 2005). Se utilizó el paquete R NbClust (Charrad et al., 2014) para determinar la K para el ACP cargando como entrada de datos la información de los tres primeros componentes. Comprobamos si existía diferenciación poblacional calculando el F_ST_ y los porcentajes de diferenciación entre y dentro de las subpoblaciones identificadas utilizando el análisis de varianza molecular (AMOVA por la sigla en inglés) en los paquetes dartR (Mijangos et al., 2022) y Poppr (Kamvar et al., 2014), respectivamente en R. Finalmente, estimamos manualmente la diversidad genética calculando la heterocigosidad observada (Ho) por genotipo y grupo genético, utilizando la metodología descrita por Berdugo-Cely et al. (2021).

**Predicción del nivel de ploidía**

Predijimos el nivel de ploidía de cada accesión utilizando la proporción de códigos de genotipo simplex y triplex (ABBB, AAAB) del conteo general a través de todos los SNPs por accesión, como lo reporto Alsahlany et al. (2019). Este método establece los umbrales para predecir la ploidía utilizando un conjunto de muestras de referencia. Para ese caso, cuando la suma de las proporciones de simplex y triplex es cercana a cero, la muestra sería diploide, y cuando la frecuencia es superior a 0,2 correspondería a tetraploide. En este estudio, validamos la precisión del método comparando la ploidía de 112 accesiones de referencia de la CCC determinadas usando la frecuencia de los códigos de genotipos SNP y los respectivos valores de ploidía obtenidos mediante conteo de cloroplastos y conteo de cromosomas reportados en tres publicaciones previas (Guevara, 2011; Uribe Gaviria, 2011; Sánchez, 2017). Para esta determinación, se implementó un análisis de varianza simple (ANOVA por la sigla en inglés) y la prueba de Tukey en el software R para encontrar diferencias significativas entre las estrategias de predicción de ploidía. Finalmente, se establecieron los parámetros para evaluar la ploidía en las muestras utilizadas en este estudio.

**Colecciones núcleo**

Se utilizó el programa Core Hunter 3 (De Beukelaer et al., 2018) para definir colecciones núcleo que representaran la diversidad genética total presente en la CCC. Se calcularon las distancias genéticas con el método modificado de Roger (MRD por la sigla en inglés) y se utilizaron para identificar las colecciones núcleo con la función de distancia A-NE. Esta función optimiza la selección de entradas para minimizar la distancia media entre cada accesión que compone la colección principal y la entrada más cercana que fue seleccionada, como se espera al crear una colección núcleo diversa. Probamos tres tamaños de colección núcleo con 20, 15 y 10% del total de muestras evaluadas en este estudio (es decir, 258, 194 y 129 entradas, respectivamente) y una colección mini-núcleo con el 3% del tamaño original (39 entradas).

Evaluamos la calidad del método A-NE para la selección de colecciones núcleos, calculando y comparando la distancia promedio obtenida con la prueba A-NE y dos métricas de distancia adicionales evaluadas por Odong et al. (2013): (1) distancia promedio entre cada entrada y la entrada vecina más cercana (E-NE), y (2) distancias genéticas promedio entre entradas (E-E). Esto se hizo para cada una de las colecciones núcleo y mini-núcleo identificadas. Para esta comparación se utilizó el paquete EvaluateCore R v.0.1.3 (Aravind et al., 2022) y dos tamaños de colección núcleo adicionales (50 y 80%).

Para validar que la representación de la diversidad genética se mantuviera en todas las colecciones y seleccionar la colección núcleo más adecuada para el mejoramiento y la investigación, se comparó la media de las medidas de heterocigosidad observada (Ho) de las colecciones núcleo y mini-núcleo con la de toda la CCC. Además, se realizó un ACP para cada colección con el fin de mostrar la distribución de las muestras seleccionadas en cada colección núcleo propuesta en comparación con la de la CCC completa. Finalmente, para la colección mini-núcleo del 3% identificada por Core Hunter, reemplazamos algunas entradas seleccionadas para que coincidieran con las accesiones dentro de la colección núcleo del 10% y así lograr que la colección del 3% este contenida en la del 10%. Para ello, a partir de la selección inicial de entradas obtenida con Core Hunter identificamos las entradas que diferían entre las colecciones mini-núcleo del 3% y núcleo del 10%. A continuación, utilizando la matriz MRD, calculamos la diferencia entre la distancia genética de cada entrada diferenciada en la mini-núcleo del 3% y las entradas diferenciadas del 10%, de esta forma se identificó la entrada del 10% con la menor diferencia de distancia genética. Por último, las entradas del 3% se sustituyeron por la correspondiente entrada identificada en la colección núcleo del 10%, este intercambio se hizo para 19 accesiones en total.

**Validación de la utilidad de las colecciones núcleo y mini-núcleo**

Validamos la efectividad de representar todo el espectro de variación fenotípica de las accesiones en las colecciones núcleo y mini-núcleo para tres rasgos diferentes: i) Peso Promedio de los Tubérculos en gramos (PPT), ii) Número de Tubérculos por Planta (NTP) y iii) Rendimiento Total de Tubérculos (RTT) en kg/planta. Para ello, utilizamos los datos de fenotipo de 846 accesiones genotipadas (206 del grupo CCC_Group_A, 494 del CCC_Group_B1 y 146 del CCC_Group_B2) que tenían evaluaciones preliminares de campo para PPT, NTP y RTT. Los datos se recopilaron entre 2013-2015. Las accesiones tienen datos para esos años, dependiendo de las accesiones evaluadas en cada temporada de campo. Debido a limitaciones financieras y de capacidad operativa, priorizamos las accesiones y los rasgos que debían evaluarse cada año. Inicialmente las accesiones de Andigenum y posteriormente las de Phureja fueron evaluadas en tres años consecutivos. Las evaluaciones fueron realizadas en una zona de altiplano del municipio de Zipaquirá, en el departamento de Cundinamarca. Los datos de campo de cada año se recolectaron de un promedio de 20 clones sembrados por surco por accesión, utilizando un diseño completo al azar para toda la CCC en campo. Así, los datos de cada año representan una repetición. Utilizamos los valores medios de las evaluaciones de todos los años por rasgo y accesión para los análisis. Esperábamos que, independientemente del tamaño, una buena colección núcleo mostrara una distribución fenotípica similar a la de las 846 accesiones. Además, analizamos si las entradas identificadas para las diferentes colecciones núcleo y mini-núcleo representaban la diversidad fenotípica de todas las accesiones, comparando la distancia genética promedio para diferentes métricas obtenidas para las pruebas A-NE, E-NE y E-E. La distancia de Gower (Pavoine et al., 2009) entre accesiones se calculó utilizando la matriz de variables fenotípicas. A continuación, al igual que en la evaluación anterior para SNP, se cargó el programa EvaluateCore R con esta información para calcular las diferentes métricas basadas en la distancia. Se utilizaron las entradas seleccionadas en las colecciones núcleo y mini-núcleo que contenían datos fenotípicos.

**RESULTADOS**

**Estructura de la población y diversidad genética**

Tras los controles y filtros de calidad, identificamos 3.586 marcadores polimórficos de nucleótido único (SNP) en todo el genoma para realizar los análisis (Tabla suplementaria S2). El análisis de la estructura de la población con las metodologías de STRUCTURE y NbClust sugirió dos y tres grupos genéticos dentro del germoplasma de la CCC, respectivamente (Figura 1A, Figura 1B y Figuras suplementarias S1A y S1B). El nivel de ploidía fue el principal factor determinante de la identificación de los grupos, seguido de la clasificación taxonómica asignada originalmente a las diferentes accesiones de la colección (Tabla 2). Los análisis de genética de poblacionales apoyaron estos resultados mostrando una estructura genética sólida y significativa para los dos (Phi=0,370; p-valor=0,001) y tres (Phi=0,359; p-valor=0,001) grupos genéticos detectados en la CCC. El ACP también mostró resultados similares (Figuras 1B y 2B). El primer y segundo componente explicaron el 19,94% y el 8,04% de la varianza de la muestra, avalando la diferenciación de las accesiones por nivel de ploidía y taxonomía con cierto nivel de mezcla.

En la clasificación taxonómica más reciente, las papas cultivadas más tradicionales y las modernas se clasifican dentro de una única especie de papa. Sin embargo, la taxonomía de Hawkes asignada originalmente a la CCC concuerda con una clara diferenciación genética inherente al germoplasma. Por lo tanto, para este trabajo, designamos las accesiones como tipos Phureja, Andigena y Tuberosum. El análisis de estructura poblacional realizado mediante STRUCTURE discriminó la CCC en dos grupos genéticos, el CCC_Group_A con accesiones principalmente diploides (2n=2x=24) y el CCC_Group_B con accesiones mayoritariamente tetraploides (2n=4x=48). El segundo análisis realizado utilizando Nbclust detectó tres grupos genéticos, el mismo grupo diploide (CCC_Group_A) identificado previamente, mientras que el grupo tetraploide se separó en dos nuevos grupos genéticos (CCC_Group_B1 y CCC_Group_B2). Como se muestra en la Tabla 2, el grupo genético CCC_Group_A contenía 248 accesiones. De ellas, el 96,4% (n=239) eran diploides y el 3,6% (n=9) eran tetraploides. A nivel taxonómico, la mayoría de las accesiones (n=137, 55,2%) eran del tipo Phureja, mientras que el 2,8% (n=7) eran Andigenum, el 1,6% (n=4) Tuberosum, el 0,4% (n=1) era *S. rybinni*, y del 39,9% (n=99) no tenemos información. Esto debido a que no se han digitalizado los registros impresos de la clasificación taxonómica asignada a estas accesiones cuando el material de la CCC fue adquirido. Por lo tanto, esta documentación se encuentra actualmente en proceso de curación. El CCC_Group_B1 cuenta con 640 accesiones, 96,7% (n=619) accesiones tetraploides y 3,2% (n=21) con ploidía desconocida. Taxonómicamente, la mayoría de las accesiones, el 81,4% (n=521), fueron Andigenum, y una proporción menor Tuberosum, el 2,7% (n=17), y Phureja, el 0,3% (n=2). El 15,6 % restante (n=100) se encuentra actualmente en proceso de clasificación taxonómica. Por último, el grupo genético CCC_Group_B2 contó con un 100% (n=273) de accesiones tetraploides. A nivel taxonómico, el 56 % (n=153) correspondió a Andigenum, el 16,8 % (n=46) a Tuberosum, el 1,5 % (n=4) a Phureja, y el 25,6 % (n=70) están pendientes de clasificación taxonómica. Finalmente, las accesiones evaluadas en este estudio que son variedades liberadas o germoplasma de programas de fitomejoramiento -algunas de las cuales forman parte de la CCC- fueron distribuidas uniformemente entre los grupos genéticos. En consecuencia, algunas son diploides y otras tetraploides.

La diferenciación genética es significativa entre los grupos genéticos CCC_Group_A y CCC_Group_B (F_ST_=0,146, valor p<0,0001). El grupo genético CCC_Group_A mostró una diferencia genética menor con el CCC_Group_B1 que con el CCC_Group_B2 (F_ST_=0,164, *p-value*<0,0001 y 0,206, *p-value*<0,0001, respectivamente), mientras que los grupos tetraploides son más similares (F_ST_=0,071, p-value<0,0001). Así, el CCC_Group_B1 presenta un nivel de diferenciación intermedio entre los grupos genéticos CCC_Group_A y CCC_Group_B2. Los resultados mostraron una elevada variación genética en el germoplasma de la CCC, confirmada por una mayor variación significativa dentro de los grupos genéticos [62,95% para la estructura genética con dos grupos (K2) y 64,08% para tres grupos (K3)] que entre grupos (37,04% para K2 y 35,91% para K3). Basándose en los valores de heterocigosidad divergente Ho para la estructura genética de dos (CCC_Group_A: 0,14±0,04 y CCC_Group_B: 0,42±0,08) y tres grupos genéticos (CCC_Group_A: 0,14±0. 04; CCC_Group_B1: 0,37±0,04, y CCC_Group_B2: 0,53±0,04), la variación genética dentro de los grupos CCC_Group_B, B2 y B1 fue mayor que la observada en el grupo CCC_Group_A (Figura 1C y 2C).

**Determinación de la ploidía**

Se utilizó una muestra de referencia de 112 accesiones para confirmar la precisión de la predicción del nivel de ploidía de las accesiones de la CCC utilizando la proporción de codificación de genotipo simplex y triples de los marcadores SNP por muestra, de acuerdo con lo reportado por Alsahlany et al. (2019). Los resultados de esta determinación de la ploidía se compararon con los obtenidos con otro método indirecto de determinación de la ploidía, el número medio de cloroplastos por célula guarda, y – con un método directo, el número de cromosomas contados en las células de la raíz, partiendo de datos publicados anteriormente (Guevara, 2011; Uribe Gaviria, 2011; Sánchez, 2017). En general, el conjunto de muestras de referencia mostró un patrón para el número de cloroplastos por célula guarda y la proporción de codificación de simplex y triplex de todos los SNPs por muestra, asociados al número de cromosomas (Figura 3 y Tabla Suplementaria S1). El nivel de ploidía basado en el recuento cromosómico, un método directo y preciso de evaluación de la ploidía, para las 112 accesiones del conjunto de referencia incluyó 83 diploides (2n=2x=24), 27 tetraploides (2n=4x=48) y dos triploides (2n=3x=36). Como era de esperar, el número básico de cromosomas del género *Solanum* (12) se duplicó de 2-4 veces según el nivel de ploidía de cada accesión (Figura 3A y Tabla Suplementaria S1). Por lo tanto, esta determinación de la ploidía se utilizó para comparar la precisión de los métodos indirectos. El conteo de cloroplastos en las células guarda coincidieron con una media de 7,6±0,83 cloroplastos/célula guarda para las accesiones diploides y de 13,2±1,06 cloroplastos/célula guarda para las accesiones tetraploides. El grupo tetraploide difirió significativamente del grupo diploide (*valor p* <0,0001). El conteo de cloroplastos en las células guarda no pudo predecir las accesiones triploides. La mayoría de las accesiones diploides (74/83, 89,2%) tenían una media de 7-8 cloroplastos/célula guarda, y unas pocas (9/83, 10,8%) estaban en una zona gris con un promedio entre 9-10 cloroplastos/célula guarda. Los tetraploides fueron más consistentes con 12-14 cloroplastos/células guarda para el 96% de las accesiones (26/27) y una en una zona gris de 10 cloroplastos/células guarda.

En el caso de utilizar la proporción de codificación de genotipos de SNP como método indirecto para evaluar la ploidía, este análisis mostró una media de 4,8%±9,04 de codificaciones de SNP con genotipos simplex y triplex para las accesiones diploides y un 27,1%±1,53 para las accesiones tetraploides. Además, el ANOVA confirmó diferencias significativas entre la media de las muestras diploides y tetraploides (*valor p* < 0,001), como se muestra en la Figura 3C y en la Tabla Suplementaria S1. Al igual que el método de conteo de cloroplastos, este método no pudo discriminar las muestras triploides. Del total de accesiones diploides basadas en el conteo cromosómico, para 77, la proporción de codificaciones de SNP simplex y triplex estuvo entre 0-15%, y para seis accesiones, la frecuencia de codificaciones de SNP simplex y triplex estuvo fuera de tipo con 24-41%. Para las muestras tetraploides, la proporción de codificaciones de genotipos SNP simplex y triplex por accesión fue exacta para 26 muestras tetraploides con valores entre el 22-42% y una muestra fuera de tipo con 0%. El desajuste entre métodos podría deberse a una mezcla de muestras entre la primera evaluación y la actual, ya que el muestreo para cada evaluación tuvo lugar en momentos distintos. A diferencia de conteo de cloroplastos y cromosomas, este desajuste no se produjo, ya que los métodos se evaluaron utilizando muestras de la misma fuente de material vegetal. Si los consideramos errores reales del método, la precisión de la predicción de ploidía utilizando codificaciones de genotipos de SNP simplex y triplex por accesión fue del 92,8% (77/83) para diploides y del 96,3% para tetraploides (26/27).

Basándonos en estos resultados, predecimos la ploidía de todas las muestras utilizadas en este estudio utilizando un umbral de 0-15% de frecuencia de codificaciones de genotipos de SNP simplex y triplex por accesión para diploides y superior al 20% para tetraploides. Se asigno una ploidía desconocida a las accesiones con una frecuencia de simplex y triplex en una zona gris del 15-20%. De las 1.291 muestras genotipadas, 239 accesiones únicas fueron diploides, 901 tetraploides y 21 desconocidas (Tabla 2). La ploidía de las 130 repeticiones biológicas incluyó 59 diploides, 70 tetraploides y una desconocida. Para 101 accesiones con repeticiones biológicas, la evaluación de la ploidía coincidió entre las repeticiones, mientras que, para 12 accesiones, algunas repeticiones no tuvieron los mismos resultados.

**Ensamblaje de las colecciones núcleo**

Utilizando el programa Core Hunter3, identificamos tres colecciones núcleo para el 10 (CCC_10), el 15 (CCC_15) y el 20% (CCC _20) del número total de muestras utilizadas en este estudio (es decir, 129, 194 y 258 entradas, respectivamente), y una colección mini-núcleo con el 3% (CCC_3) del tamaño original (39 entradas). Se confirmó la precisión del algoritmo A-NE utilizado para identificar colecciones núcleo que representan diversidad genética. Se observaron valores más bajos de la distancia promedio para A-NE en las colecciones núcleo y mini-núcleo identificadas con esta métrica. Mientras que, por el contrario, las mismas entradas en las distintas colecciones núcleo tenían una distancia media con tendencia a los valores máximos óptimos propios de las pruebas de distancia E-E o E-NE (Figura 4). Del mismo modo, como era de esperar, cuanto mayor es el tamaño de la colección, menor es la distancia media A-NE, ya que cada accesión se representa a sí misma en el conjunto de la colección. A continuación, comparamos la representación de la diversidad genética en las colecciones identificadas mediante el método A-NE. No encontramos diferencias entre las colecciones núcleo y mini-núcleo en comparación con la colección primaria utilizando el ACP y la Ho para dicha comparación. El ACP mostró que todos los tamaños de colección núcleo y mini-núcleo representaban un patrón similar de distribución de la diversidad genética (Figura 5 A-D) comparable al de la colección completa (Figura 1B y 2B). Para la colección completa (CCC_100), la media de Ho fue 0,33 (±0,13), 0,36 (±0,12) para la colección núcleo de tamaño 20% (CCC_20), 0,38 (±0,12) para la colección núcleo del 15% (CCC_15) y 0,38 (±0,13) para la colección núcleo del 10% (CCC_10), mientras que la Ho para la colección mini-núcleo del 3% (CCC_3) fue 0,37 (±0,13). En general, la media de Ho fue mayor en las colecciones núcleo (Ho = 0,36-0,38) en comparación con toda la CCC (Ho = 0,33), y la distribución de la densidad de Ho de las accesiones de cada colección sigue patrones similares a los de la CCC y estaban contenidas en ella (Figura 6A y Tabla 3). Por lo tanto, proponemos una colección núcleo del 10% para la investigación y el mejoramiento en AGROSAVIA. La colección núcleo del 10% tiene un tamaño pequeño adecuado que ya captura la diversidad total de la CCC y tiene un número manejable de accesiones para proyectos de investigación y mejoramiento. La proporción de muestras en los tres grupos genéticos (CCC_Group_A, CCC_Group_B1 y CCC_Group_B2) fue del 24%, 53% y 24% para todas las muestras genotipadas y del 15%, 52% y 33% para la colección núcleo del 10%.

Por último, utilizamos los datos fenotípicos disponibles de tres rasgos agronómicos (PPT, NTP y RTT) para 846 accesiones genotipadas de la CCC con el fin de validar la representación de la variación fenotípica en las colecciones núcleo y mini-núcleo. La estrategia de validación demostró que las 846 accesiones genotipadas y fenotipadas tenían un rango de distribución similar de valores para los estadísticos centrales y de dispersión en toda la colección (CCC_100) y a través de los diferentes tamaños de núcleo (CCC_20-10) y mini-núcleo (CCC_3) para los tres diferentes rasgos (Figura 6 B-D y Tabla 3). En contraste con los valores de diversidad genética en términos de Ho entre colecciones, con una tendencia a aumentar desde la colección principal al tamaño más pequeño de la colección central (Figura 6A), para el rasgo de PPT, las medias en las colecciones centrales disminuyeron (CCC_20 = 18,27 g - CCC_3 = 15,54 g) con respecto a la CCC completa (CCC_100 = 23,04 g) (Tabla 3). Sin embargo, los rasgos NTP (CCC_20 = 11,59 tubérculos - CCC_3 = 14,99 tubérculos; Figura 6C y Tabla 3) y RTT (CCC_20 = 0,73 kg/planta - CCC_3 = 0,92 kg/planta; Figura 6D y Tabla 3) presentaron una tendencia similar en valores medios que Ho; aquí, los valores medios para cada colección núcleo fueron mayores que en toda la CCC (en CCC_100: NTP = 11,64 tubérculos y TTY = 0,77 kg/planta).

Además, comparamos el comportamiento de tres métricas basadas en distancias utilizadas para la identificación de colecciones núcleo usando datos fenotípicos, esto para validar la calidad de las colecciones núcleo y mini-núcleo identificadas con marcadores SNP y la métrica A-NE (Figura 7). Como se observó para los datos genotípicos, se obtuvieron valores pequeños para la distancia media A-NE para los diferentes tamaños de colección núcleo. Aunque las distancias medias para las métricas E-NE y EE fueron mayores que A-NE, no mostraron una fuerte maximización de los valores para tamaños de colección pequeños. Como era de esperar, los valores medios de A-NE disminuyeron en la misma medida en que el tamaño de la colección núcleo se acercaba al tamaño de la colección completa.

**Descripción de las colecciones núcleo**

Las colecciones núcleo del 10% y mini-núcleo del 3% contienen accesiones de diferentes grupos taxonómicos y orígenes geográficos (Tabla suplementaria S1). De las 129 accesiones de la colección núcleo contenidas en la colección del 10%, 82 accesiones son *S. tuberosum* subsp. *andigenum*, 18 son *S. phureja*, 17 son *S. tuberosum* subsp. *tuberosum*, y 12 están actualmente en proceso de curación. Esta colección núcleo del 10% contiene 70 accesiones de diez sitios de colectan en Colombia, la mayor representación de los departamentos de Nariño (22), Boyacá (14), y Cundinamarca (14), seguido por Cauca (6), Antioquia (5), Valle del Cauca (5), y una accesión de Caldas, Quindío, Santander, y Tolima. Además, la colección núcleo cuenta con accesiones de Perú (7), Bolivia (3), Ecuador (2), México (2), EE.UU. (2), Países Bajos (2) y Alemania (2). Treinta y nueve de estas accesiones son de origen desconocido. De las 39 accesiones de la colección mini-núcleo (3%), 25 son *S. tuberosum* subsp. *andigenum*, seis son *S. phureja*, dos son *S. tuberosum* subsp. *tuberosum*, y seis aún no han sido identificadas y están en proceso de curación. En cuanto a su procedencia, la colección mini-núcleo cuenta con 22 accesiones de Colombia recolectadas en siete departamentos: Nariño, Cundinamarca, Boyacá, Antioquia, Cauca, Quindío y Valle del Cauca. La colección mini-núcleo tiene cuatro accesiones de otros países, dos de Perú, una de México y una de los Países Bajos. Finalmente, la colección mini-núcleo tiene 13 accesiones de origen desconocido.

**DISCUSIÓN**

Nuestro estudio genotipó el 90,9% (1141/1255) de la colección clonal de la CCC y una representación de las variedades liberadas. Estos datos genómicos nos permitieron estudiar la estructura de la población y la diversidad genética, evaluar la ploidía y definir colecciones núcleo y mini-núcleo que sean representativas de la diversidad genética de toda la CCC. A continuación, discutimos los resultados y cómo podemos usarlos para curar y mejorar la colección de papa colombiana de acuerdo con la Estrategia Global de Conservación para la papa (Nagel et al., 2022).

**Estructura poblacional y diversidad genética**

El patrón de estructura y diversidad genética poblacional observado sugiere que las accesiones de papa conservadas en la CCC se ajustan a los patrones evolutivos de selección agrícola que separaron las variedades criollas diploides de las tetraploides, y éstas de la papa cultivada más moderna (*Solanum tuberosum* L). Según nuestro estudio anterior que utilizó 809 accesiones (Berdugo-Cely et al., 2017), la CCC tiene una estructura poblacional que contiene dos grupos genéticos principales de accesiones mayoritariamente diploides y tetraploides representados en general por los tipos Phureja y Andigenum, respectivamente (CCC_Group_A y CCC_Group_B). Este estudio corroboró este patrón, y más aún separó el grupo genético tetraploide en dos grupos genéticos bien diferenciados (CCC_Group_B1 y CCC_Group_B2). El grupo CCC_Group_B1 tiene un mayor número de accesiones y niveles de mezcla que los otros dos grupos genéticos. La mayoría de las accesiones son de tipo Andigenum, probablemente con una ascendencia más primitiva que CCC_Group_B2. Por lo tanto, las accesiones de Andigenum que dieron lugar a un grupo genético CCC_Group_B2 más diferenciado y divergente, probablemente provienen de una presión de selección más reciente por parte de agricultores y mejoradores para algunos rasgos modernos. Un mayor número de accesiones de Tuberosum también se encontraban en este grupo CCC_Group_B2, apoyando la hipótesis de que este grupo sufrió una presión de selección sustancial. Esto también concuerda con el hecho de que la mayoría de las accesiones de Tuberosum en el CCC son variedades modernas adquiridas como parte de los esfuerzos de mejoramiento de papa en Colombia (Moreno y Valbuena, 2006). En papa se han reportado diferencias en los patrones de selección agrícola. Los cultivares modernos norteamericanos y tradicionales Andinos compartieron solo el 14-16% de los genes bajo selección; por lo tanto, la adaptación de Andigenum de tierras altas y Tuberosum de tierras bajas tuvo diferentes estrategias de selección (Hardigan et al., 2017). Además de esta conclusión, consideramos que las prácticas modernas de mejoramiento en América del Norte y Europa también fortalecieron la diferenciación entre la papa moderna de América del Norte y las variedades locales Andinas. Los grupos CCC_Group_B1 y CCC_Group_B2 también mostraron divergencia de Phureja, probablemente debido a la poliploidización, un rango de distribución más amplio y la presión de selección para algunos rasgos culinarios o de cultivo. En general, las papas han sido seleccionadas por los agricultores tradicionales ancestrales y, más recientemente, por los fitomejoradores por el tamaño del tubérculo, el contenido de carbohidratos y glicoalcaloides en los tubérculos, la adaptación al fotoperíodo y la fertilidad sexual reducida (Hardigan et al., 2017). Los resultados de los estudios de diversidad de la CCC apoyan la divergencia entre accesiones diploides y tetraploides de los tipos Phureja, Andigenum y Tuberosum, probablemente mediada por patrones de selección.

La CCC es una de las colecciones clonales de papas nativas de América Latina que puede compararse con la colección del Centro Internacional de la Papa (CIP) en Perú, con alrededor de 4.500 accesiones de variedades locales (Ellis et al., 2020). El análisis de diversidad genética de parte de la colección de papa del CIP concordó con los resultados de nuestro análisis de los patrones de diferenciación genética de los grupos Phureja y Andigenum observados en la CCC. Así, Andigenum tiene un número más significativo de accesiones, dosis genética y diversidad genética. Por el contrario, Phureja es más homogéneo genéticamente con un menor número de accesiones (Ellis et al., 2018). Según lo documentado por diferentes autores, la diversidad genética de las papas tetraploides nativas del grupo Andigenum tiene mayor cobertura geográfica y diversidad en comparación con el grupo Phureja distribuido en un área más restringida (Hawkes, 1990; Huamán y Spooner, 2002; Spooner et al., 2010; Bradshaw, 2021); lo que sugiere que la poliploidía dio ventajas de rendimiento, robustez y adaptación a diferentes ambientes en el altiplano de los Andes. Aunque los diploides de Phureja también se derivan de *S. stenotomum*, fueron seleccionados debido a la falta de dormancia del tubérculo, ciclos de cultivo más cortos y adaptabilidad a valles más cálidos, bajos y orientales de los Andes. Además de la adopción menos cosmopolita de la papa Phureja diploide, algunos niveles de autocompatibilidad también podrían haber contribuido a la homogeneidad genética de este grupo. Aunque la naturaleza autoincompatible de las papas diploides está ampliamente aceptada, informes recientes han revelado la existencia de fuentes de autocompatibilidad en las papas cultivadas (Kaiser et al., 2021). En particular, el clon 1S1 de Phureja ha sido identificado como fuente de autocompatibilidad, lo que nos lleva a suponer que la autocompatibilidad contribuye a la diversidad genética de Phureja (ibid). En resumen, tanto la colección de papa del CIP como la CCC mostraron patrones similares de diferenciación de la diversidad genética para las accesiones de Phureja y Andigenum.

En diferentes estudios de estructura poblacional y diversidad genética de colecciones de bancos de germoplasma de cultivares tradicionales y programas de mejoramiento de papa, la discriminación del material se ha soportado en caracteres de ploidía, taxonomía y características de selección de mejoramiento (Hirsch et al., 2013; Berdugo-Cely et al., 2017; Hardigan et al., 2017; Deperi et al., 2018; Ellis et al., 2018; Pandey et al., 2021). En general, se observa que los estudios de diversidad genética de germoplasma procedente de bancos de germoplasma han mostrado que la discriminación entre accesiones está asociada principalmente a la ploidía y a la taxonomía (Berdugo-Cely et al., 2017, 2021; Hardigan et al., 2017; Ellis et al., 2018). Esta discriminación concuerda con nuestros resultados, en los que la ploidía y la taxonomía explicaron los grupos genéticos identificados. Por el contrario, los estudios de diversidad genética de germoplasma procedente de programas de fitomejoramiento han demostrado que la discriminación de las accesiones depende de la selección por rasgos de calidad asociados a diferentes tipos de mercado de la papa, lo que indica que la diversidad está moldeada por la fuerte presión de selección de los rasgos de mejoramiento (Hirsch et al., 2013; Deperi et al., 2018; Pandey et al., 2021). En general, los estudios sobre la estructura de la población y la diversidad genética de la papa mostraron que la introgresión de rasgos a partir de germoplasma silvestre y la presión de selección de la mejora contribuyeron a aumentar la heterocigosidad y la diversificación de los linajes cultivados.

**Determinación de la ploidía**

Este estudio utilizó la frecuencia de las codificaciones de simplex y triplex de todos los SNPs en cada accesión como predictor del nivel de ploidía, siguiendo el reporte de Alsahlany et al. (2019). Esta metodología es posible porque el genotipado de SNPs mediante la tecnología de microarreglos en perlas de Illumina permite identificar la dosificación de marcadores bialélicos en una especie autotetraploide como es la papa. Alsahlany et al. (2019) propusieron evaluar la ploidía basándose en la frecuencia de los marcadores SNP simplex y triplex (ABBB, AAAB), en los que un alelo estaría en mayor proporción que el otro. Este enfoque es objetivo porque las accesiones diploides sólo tendrían dos alelos, lo que daría la misma proporción para cada uno; por lo tanto, hay una probabilidad baja o nula de detectar una codificación simplex o triplex en los SNP de un diploide. Los genes parálogos o los desfases entre el ADN de la muestra y la sonda del SNP podrían explicar la detección de codificaciones simplex o triplex en diploides, pero la probabilidad de que esto ocurra es baja.

En este estudio, la frecuencia de codificaciones simplex y triplex de los SNP identificadas en el conjunto de muestras de referencia varió en comparación con el reportado por Alsahlany et al. (2019); los tetraploides tienen una proporción del 27,1%±1,53 en comparación con el 36%±14, y los diploides del 4,8%±9,04 en comparación con el 2%±1. El umbral de separación para predecir la ploidía de nuestras muestras fue <15% para diploides y >20% para tetraploides. Las accesiones con frecuencias entre 15 y 20% se consideraron desconocidas; esta clasificación fue ajustada en comparación con la amplia separación reportada por Alsahlany et al. (2019). La frecuencia de simplex/triplex fue de hasta el 4% para la mayoría de las muestras diploides (263/297, 88,6%), y unas pocas muestras estuvieron entre el 9-15% (34/297, 11,4%). La diversidad genética de esta colección probablemente interfirió con la hibridación del ADN durante el genotipado de los SNP para esas muestras y afectó a la proporción de codificaciones de genotipos de SNP simplex y triplex. Por el contrario, Alsahlany et al. (2019) utilizaron germoplasma de mejoramiento Norteamericano similar a las variedades utilizadas para desarrollar la matriz SNP de papa (Hamilton et al., 2011). Aunque para unas pocas muestras, los parámetros de clasificación utilizados para determinar la ploidía de las accesiones en la CCC basados en la frecuencia de codificaciones de SNP simplex y triplex fueron ajustados, esta metodología permitió estimar la ploidía en la CCC.

Los valores de heterocigosidad estimados para muestras de papa genotipadas con el mircroarreglo Infinium de Illumina para papa también se han relacionado con los niveles de ploidía. Ellis et al. (2018) reportaron que cada grupo de ploidía de un panel de 500 muestras de variedades locales de papa se asoció con un patrón de porcentaje de heterocigosidad. Sin embargo, también detectaron que la heterocigosidad se solapa en rangos estrechos, dificultando la separación. Encontraron una mayor heterocigosidad en el germoplasma tetraploide (>30%) en comparación con los diploides (<20%), y los triploides tienen un 20-30% de heterocigosidad. En el presente estudio, encontramos que los rangos de heterocigosidad para las muestras del conjunto de referencia, excluyendo los errores potenciales, eran del 10-23% para los diploides, del 13% para los triploides y del 33-58% para los tetraploides. Sin embargo, había una distribución de datos continua de 9-66% de heterocigosidad para las muestras completas sin una separación clara. Esperábamos principalmente diploides, tetraploides y un bajo número de triploides en la CCC (Figuras 2C y 3C). Estos resultados mostraron que los umbrales de separación deben definirse para cada caso en función del germoplasma evaluado. Del mismo modo, la frecuencia de las codificaciones de simplex y triplex de todos los SNP en una accesión es un método mucho mejor para predecir el nivel de ploidía que los valores de heterocigosidad.

Un conjunto de referencia de muestras con información sobre ploidía generada por otros métodos indirectos (conteo de cloroplastos por célula guarda o citometría de flujo) y/o directos (conteo de cromosomas) definiría mejor la separación de umbrales en la evaluación de la ploidía. En nuestro caso, comparamos los datos de las codificaciones de los SNP, el número de cloroplastos/células guarda y el conteo de cromosomas. El umbral de separación para el número de cloroplastos/células guarda (6-8 para diploides y 12-14 para tetraploides) fue coherente con reportes anteriores (Rasmussen y Rasmussen, 1995; Gebhardt et al., 2006; Ordoñez et al., 2014; Alsahlany et al., 2019). Aunque la determinación de la ploidía utilizando únicamente la codificación de los SNP es válida, recomendamos confirmar esta evaluación con una técnica de bajo costo y eficiente en el tiempo, como el conteo de cloroplastos en células guarda.

**Colección principal**

Propusimos el subconjunto del 10% como la colección núcleo porque ya capturaba la representación de la diversidad total de la CCC. La colección núcleo, reducida, pero altamente diversa, tiene un número más manejable y versátil de accesiones para el descubrimiento de rasgos, evaluaciones y uso en el mejoramiento genético y de cultivos de papa en Colombia. La representación de la diversidad fenotípica para tres características agronómicas (PPT, NTP y RTT) dentro de las colecciones núcleo y mini-núcleo confirmó su uso potencial. En general, la evaluación de la CCC en busca de fuentes de nuevos alelos para diferentes rasgos ha llevado varios años debido al tamaño de esta colección. Ahora, se puede hacer un tamizado más rápido, y la colección mini-núcleo, con un tamaño aún más pequeño, es una alternativa cuando la evaluación de la colección núcleo no es factible.

Como era de esperar, tras eliminar la redundancia en la colección, la diversidad genética medida utilizando la media de Ho aumentó en las colecciones núcleo y mini-núcleo. Resultados similares han sido reportados para colecciones núcleo de papa recientemente desarrolladas (Bamberg y del Río, 2004; Pandey et al., 2021). El análisis ACP también mostró que la dispersión de la muestra en los gráficos de dispersión tiene una distribución similar a través de los tamaños de las colecciones núcleo. Asimismo, el análisis del comportamiento de las distancias promedio para las métricas de distancia A-NE, EE y E-NE utilizadas para identificar colecciones núcleo confirmó que la métrica A-NE es robusta para obtener una representación uniforme del espectro genético original, tal y como proponen Odong et al. (2013). El algoritmo A-NE tiene como objetivo obtener una distancia media pequeña entre la accesión y la entrada más cercana dentro de la colección núcleo para toda la colección, lo que garantiza la representatividad de todas las accesiones en la colección núcleo. Por el contrario, los métodos E-E y E-NE maximizan las distancias genéticas medias entre las entradas y la entrada más próxima para identificar las colecciones núcleo que representan valores extremos o una distribución, respectivamente. El promedio A-NE también disminuyó a medida que aumentaba el tamaño de la colección porque, como era de esperar, la máxima representación de cada accesión por una entrada es ella misma. Los promedios de A-NE de las colecciones núcleo y mini-núcleo identificadas siguieron el comportamiento esperado utilizando datos fenotípicos.

El tamaño seleccionado de la colección núcleo del 10% está dentro del rango del 5-20% de tamaños reportados para colecciones núcleo y corresponde con el tamaño más recomendado (Van Hintum et al., 2000). Presentaremos esta colección núcleo a los fitomejoradores y científicos de AGROSAVIA para obtener sus comentarios y contribuciones con el fin de garantizar que las entradas seleccionadas sean las más adecuadas para la investigación y el mejoramiento. Si fuera necesario, las accesiones de interés para la investigación y la mejora o con largos registros históricos de evaluación podrían añadirse a la colección núcleo, esto se haría sustituyendo las entradas seleccionadas inicialmente que tengan una la distancia genética similar a las nuevas accesiones.

**Uso práctico de la determinación de la diversidad y la a ploidía en la gestión de la CCC en AGROSAVIA**

La CCC de papa conservada en AGROSAVIA ha sido curada con el apoyo de estudios de diversidad y determinación de ploidía. La CCC clonal de papa ha estado bajo la administración de AGROSAVIA desde 1994. En ese entonces, en la colección manejada por el curador anterior se categorizaron las accesiones en diferentes grupos: Phureja, Andigenum, Tuberosum, "Guata", "Chaucha" y desconocida. Esta clasificación se basa en una combinación de patrones morfológicos específicos de la clasificación taxonómica de Andigenum, Phureja y Tuberosum, y una clasificación no taxonómica sino etnobotánica dada a algunas accesiones de germoplasma nativo o de mejoramiento, las categorías son conocidas como "Guata" y "Chaucha". Esta categorización de AGROSAVIA ha sido ajustada para cada accesión durante el proceso de curaduría de la CCC para asignar y/o verificar la clasificación de acuerdo con la clasificación taxonómica de Hawkes, (1990). Inicialmente, la colección fue caracterizada morfológicamente para documentar los descriptores discriminatorios en la CCC y las características de cada accesión. Luego, la digitalización de los datos de pasaporte con la información de la clasificación taxonómica originalmente asignada, el análisis de la diversidad genética y la determinación de la ploidía apoyaron la curación de la clasificación. En años anteriores, se utilizaron descriptores morfológicos y análisis de diversidad genética para reasignar algunas accesiones conocidas como "Chauchas" al grupo Phureja. Para las accesiones evaluadas en este estudio, encontramos que la clasificación "Guata" corresponde a accesiones que son en su mayoría tetraploides (83/88, 94,3%), y con base en pocos datos de pasaporte recuperados (23/88), las accesiones también corresponden a *S. tuberosum* subsp. *andigenum*. La asignación a grupos genéticos, la ploidía, la morfología y los registros históricos deben tenerse en cuenta para reasignar la clasificación taxonómica. Algunas accesiones diploides pueden ser Andigenum, y algunas accesiones tetraploides Phureja. En las papas, la ploidía del germoplasma puede incrementarse debido a cruces en los que intervienen gametos 2n (poliploidización) o reducirse mediante polinización con genotipos inductores de haploidía (haploidización). Las accesiones de Phureja son principalmente diploides, pero pueden encontrarse triploides y tetraploides en una proporción baja (Ghislain et al., 2006). Del mismo modo, las accesiones de Andigenum son principalmente tetraploides; sin embargo, podrían haberse generado dihaploides como parte de las estrategias de los programas de mejora. Por lo tanto, la descripción morfológica, los datos de pasaporte, el análisis de la diversidad y la determinación de la ploidía deben continuar apoyando la curación de esta colección.

En Colombia y Ecuador, las comunidades indígenas y los agricultores tradicionales clasifican los cultivares nativos entre "Chauchas" y "Guatas". En general, los cultivares con un ciclo de cultivo corto y papa de brotación temprana se conocen como "Chauchas", que significa "suave o fácil", y los cultivares con un ciclo de cultivo anual y color de pulpa del tubérculo blanco como "Guatas", palabra quechua que significa "papa de un año" (Monteros-Altamirano, 2017; Rosero Alpala et al., 2020). Desde el punto de vista taxonómico, la clasificación indígena o etnobotánica de "Chaucha" contiene germoplasma que corresponde principalmente a *S. phureja* siguiendo la clasificación anteriormente aceptada de Hawkes (1990), y en menor proporción a *S. chaucha*, mientras que las "Guatas" se asocian principalmente a *S. tuberosum* subsp. *andigenum* (Monteros-Altamirano, 2017; Navarro et al., 2010; Rosero Alpala et al., 2020). Esta homologación de la clasificación etnobotánica y taxonómica concuerda con los resultados obtenidos en la curación de la colección de la CCC, por lo que esta información etnobotánica podría apoyar la clasificación taxonómica (Figura 1A).

Parte del proceso de curación consiste en mantener toda la información relacionada con el germoplasma organizada por atributos o categorías. La base de datos debe tener información sobre nombres comunes, clasificación taxonómica basada en diferentes autores (Hawkes, Spooner), estado biológico (silvestre, nativo/tradicional/criollo/local y germoplasma de mejoramiento/investigación) y clasificación etnobotánica (Chaucha y Guatas). En cuanto a las diferentes clasificaciones taxonómicas, es importante mantener la información de la clasificación de Hawkes, que facilita el manejo y uso del germoplasma en los programas de conservación y mejoramiento. Aunque la clasificación basada en Spooner et al. (2007) que reagrupa en una sola especie cuatro especies anteriores (*S. phureja*, *S. tuberosum* subsp. *andigenum*, *S. stenotomum*, y *S. chaucha*) está bien documentada, un conjunto de descriptores morfológicos, la duración del ciclo de vida de las plantas, la adaptación al fotoperiodo, y el tipo de citoplasma respaldan que existen los niveles de diferenciación propuestos en la clasificación anterior (Hawkes, 1990; Spooner et al., 2014). Si esta distinción no se conoce, los científicos y los mejoradores pueden encontrarse con problemas en rasgos de fertilidad, dormancia, atributos del tubérculo, tuberización y madurez.

**Perspectivas futuras para la gestión de la CCC**

En este estudio se genotipó con éxito el 90,9% de la colección clonal de la CCC de papa y una representación de variedades de liberadas. Este resultado es un logro sobresaliente teniendo en cuenta el tamaño de esta colección (2499 accesiones de papa cultivada y silvestre) y su importancia para la región. Además, nos interesa que esta colección se convierta en una referencia regional. Por lo tanto, se recomienda alinear la estrategia de conservación de la CCC con las diez acciones sugeridas para mejorar la estrategia global de conservación de los recursos genéticos de la papa propuestas por varias instituciones y organizaciones (Nagel et al., 2022). Algunas de las acciones que se pueden llevar a cabo después de este estudio incluyen encontrar duplicados, clarificar la posible mezcla de accesiones expuesta en este estudio, determinar la ploidía utilizando el conteo de cloroplastos en células guarda, verificar la taxonomía y el estado biológico para aquellas accesiones en proceso de curación, completar la digitalización de la documentación y centralización de todos los datos históricos y nuevos de evaluación en la plataforma GRIN-Global, priorizar el uso de la colección núcleo para futuras evaluaciones en los experimentos de campo y recolección de datos, y completar la información de evaluación a factores bióticos y abióticos de las accesiones núcleo que está faltando de evaluaciones previas. Esta colección tiene datos de pasaporte, caracterización morfológica y perfiles genéticos que son útiles para comparar la colección *in vitro* y de campo para verificar la identidad del germoplasma. Ellis et al. (2018) mostraron que con el tiempo podría producirse cierta mezcla de accesiones en los bancos de germoplasma. Por lo tanto, a través de un proceso de verificación de identidad, la colección puede revisarse, documentarse y organizarse para corregir errores y mitigar errores futuros mediante la digitalización de datos, la implementación de códigos de barras y estándares de gestión de calidad desarrollados para bancos de germoplasma.

Los datos de genotipado generados en este estudio son comparables con los de otras colecciones genotipadas con la misma tecnología, como la colección del CIP. Esta comparación ayudará a identificar marcas o patrones de selección, materiales con potencial uso en mejora genética, duplicidad, vacíos genéticos, homologar las accesiones entre las colecciones y a evaluar la representación de la diversidad de la CCC dentro de la colección más extensa del mundo de papas nativas. Este documento también está disponible en español para continuar con esta discusión dentro del país y la región (Archivo S1).

**AGRADECIMIENTOS**

Agradecemos especialmente a L.P. Delgadillo por apoyar las extracciones de ADN de una parte de las accesiones de papa utilizadas en este estudio. Gracias a B. Coronel y R.T. Torres por recolectar hojas de algunas accesiones de la CCC en el campo. También agradecemos a R.T. Torres por localizar y organizar todos los datos de pasaporte de la CCC utilizando los libros más antiguos, sistematizados a mano. Agradecemos también a M.S. Cerón por compartir los estudios de la ploidía en la CCC. Agradecemos al Laboratorio Molecular del C.I. Tibaitatá de AGROSAVIA por su apoyo en la genotipificación de las accesiones de la CCC. Finalmente, agradecemos al antiguo curador, Iván Valbuena, por su dedicación a la CCC que motivó este trabajo y a Olga Spellman, Escritora de Ciencia, Editora y Coordinadora del Servicio de Redacción Científica de la Alianza (Alianza de Bioversity International y CIAT) por el inglés y la corrección de este manuscrito.

**CONTRIBUCIONES DE LOS AUTORES**

RY, JABC, ICS, y PHRH concibieron el estudio. JABC, ZLP y PHRH organizaron las bases de datos del CCC y diseñaron el análisis. ZLP e ICS recolectaron hojas de accesiones seleccionadas. JABC e ICS trabajaron en el laboratorio. JABC y PHRH realizaron el análisis, incluidos los gráficos descriptivos. NMC dirigió la redacción del manuscrito y realizó parte de los análisis. Todos los autores escribieron y aprobaron la versión definitiva.

**DECLARACIÓN DE DISPONIBILIDAD DE DATOS**

Este artículo publicado y el material suplementario incluyen todos los datos generados o analizados durante este estudio. Los datos SNP utilizados en este manuscrito se proporcionaron en los datos suplementarios (Material Suplementario Tabla S2). Los datos se obtuvieron de la matriz SNP de la papa. Para más información, diríjase al autor correspondiente.

**FINANCIACIÓN**

Esta investigación fue apoyada por el Ministerio de Agricultura y Desarrollo Rural de Colombia bajo los fondos TV19-21 en el proyecto "Diseño e implementación de una Plataforma de genotípificación para el Banco de Germoplasma Vegetal de Colombia conservado por AGROSAVIA" con código 1001386.

**Conflictos de intereses**

Todos los autores declaran no tener conflicto de interés.

**Consentimiento para publicación**

Todos los autores consienten la publicación de este estudio.

**LEYENDA DE LAS FIGURAS**

**Figura 1**. Estructura poblacional y análisis de diversidad genética de la Colección Central Colombiana (CCC) de papa para dos grupos genéticos. **(A)** Diagrama de barras del análisis de estructura genética para dos grupos genéticos (K=2) identificados utilizando el software STRUCTURE y designados como CCC_Group_A y CCC_Group_B (línea inferior). La clasificación del estado biológico del germoplasma de AGROSAVIA (CCC_Group) se indica en la línea superior (Andigena, Phureja, Tuberosum, "Guata" y Desconocido), **(B)** Diagrama de dispersión del Análisis de Componentes Principales (ACP) codificado por colores según el grupo genético y conformado siguiendo la clasificación del estado biológico de AGROSAVIA (CCC_Group), y **(C)** Diagrama de cajas con la distribución (la caja representa el segundo y tercer cuartil, la línea cruzada la mediana y la línea vertical el rango de datos) de la heterocigosidad observada (Ho) para cada grupo genético.

**Figura 2**. Estructura de la población y análisis de la diversidad genética de la Colección Central Colombiana (CCC) de papa para tres grupos genéticos. **(A)** Diagrama de barras del análisis de la estructura genética para tres grupos genéticos (K=3) identificados mediante el paquete R NbClust y designados como CCC_Group_A, CCC_Group_B1 y CCC_Group_B2 (línea inferior). La clasificación del estado biológico del germoplasma AGROSAVIA (CCC_Group) se indica en la línea superior (Andigena, Phureja, Tuberosum, "Guata" y Desconocido), **(B)** Diagrama de dispersión del Análisis de Componentes Principales (ACP) codificado por colores según el grupo genético y conformado siguiendo la clasificación del estado biológico AGROSAVIA (CCC_Group), y **(C)** Diagrama de cajas con la distribución (la caja representa el segundo y tercer cuartil, la línea cruzada la mediana y la línea vertical el rango de datos) de la heterocigosidad observada (Ho) para cada grupo genético.

**Figura 3**. Comparación de los métodos directo e indirecto de determinación de la ploidía en un conjunto de referencia de accesiones de la Colección Central Colombiana de la Papa (CCC). En el eje y están las escalas de medición de los métodos directo e indirecto: **(A)** conteo de cromosomas en las puntas de las raíces, **(B)** conteo de cloroplastos por célula guarda, y **(C)** proporción de codificación de genotipos polimórficos de nucleótido único (SNP) simplex y triplex (ABBB, AAAB) por muestra, comparados con el eje x que indica la ploidía obtenida con el método directo de evaluación de la ploidía. Los valores p corresponden a la significancia estadística según la ANOVA, mientras que las letras diferentes corresponden a los resultados de la prueba de separación de medias Tukey.

**Figura 4.** Evaluación cualitativa del promedio de la métrica *Accession to Nearest Entry* (A-NE) utilizada para la identificación de colecciones núcleo que representan diversidad genética y la calidad de las colecciones núcleo. La distancia promedio de las pruebas (ejes y) para tres métricas de distancia A-NE (distancia media de Accesión a Entrada más Cercana), E-E (distancia media de Entrada a Entrada), y E-NE (distancia media de Entrada a Entrada Más Cercana) calculadas utilizando las entradas de las colecciones núcleo identificadas con la prueba A-NE y marcadores de nucleótido único (SNP) para seis tamaños de colección núcleo: 3, 10, 15, 20, 50 y 80% del total de muestras genotipadas en este estudio (ejes x).

**Figura 5**. Distribución de los Análisis de Componentes Principales (ACP) de las entradas seleccionadas en tres colecciones núcleo y una mini-núcleo evaluadas para la CCC de papa. **(A)** Colección núcleo de tamaño 20% (CCC_20), **(B)** Colección núcleo de tamaño 15% (CCC_15), **(C)** Colección núcleo de tamaño 10% (CCC_10) y **(D)** Colección mini-núcleo de tamaño 3% (CCC_3). La información CCC_K3 en A-D corresponde a los tres grupos genéticos sugeridos por el análisis de la estructura de la población de la CCC utilizando el paquete R NbCluster.

**Figura 6**. Distribución fenotípica para tres características agronómicas diferentes comparada con la distribución de la diversidad genética a través de las colecciones núcleo y mini-núcleo propuestas para la Colección Central Colombiana (CCC) de papa. Distribución de valores para **(A)** heterocigosidad observada (Ho), **(B)** peso promedio de tubérculos en g (PPT), **(C)** número de tubérculos por planta (NTP), y **(D)** rendimiento total de tubérculos en Kg/planta (RTT) para toda la CCC (CCC_100), y tres tamaños de colección núcleo: 20% (CCC_20), 15% (CCC_15), y 10% (CCC_10) y una colección mini-núcleo con un tamaño del 3% (CCC_3).

**Figura 7**. Evaluación de la calidad de la colección núcleo utilizando tres diferentes métricas de distancia para la identificación de colecciones núcleo y datos fenotípicos. La distancia promedio de la prueba (eje y) para tres métricas de distancia A-NE (distancia media de Accesión a Entrada más Cercana), E-E (distancia media de Entrada a Entradas) y E-NE (distancia media de Entrada a Entrada Más Cercana) calculadas utilizando la distancia fenotípica entre las entradas de las colecciones núcleo identificadas con la prueba A-NE y los marcadores genómicos para seis tamaños de colección: 3, 10, 15, 20, 50 y 80% del total de muestras genotipadas en este estudio (ejes x).

**Tabla 1**. Origen geográfico de las accesiones de la Colección Central Colombiana de papa conservadas en la Corporación Colombiana de Investigación Agropecuaria (AGROSAVIA).

**Tabla 2**. Características taxonómicas y de ploidía de tres grupos genéticos identificados en la Colección Central Colombiana (CCC) de papa con base en los análisis de estructura poblacional y diversidad genética.

**Tabla 3**. Medidas de tendencia central (media y mediana) y dispersión [coeficiente de variación (CV), desviación estándar (Std Dev), valores mínimo (min) y máximo (Max)] para la Heterocigosidad Observada (Ho), y Peso Promedio de Tubérculos (PPT), Número de Tubérculos por Planta (NTP) y Rendimiento Total de Tubérculos (RTT) evaluados en la Colección Central Colombiana (CCC_100) de papa y Colecciones Núcleo para el 20% (CCC_20), 15% (CCC_15), 10% (CCC_10) y 3% (CCC_3) de la CCC propuesta en este estudio.

**MATERIAL SUPLEMENTARIO**

**Figuras**

**Figura suplementaria S1**. Número de conglomerados genéticos sugeridos por **(A)** STRUCTURE Harvester **(B)** y la prueba NBClust R-Package para 1.291 muestras genotipadas de la Colección Central Colombiana (CCC) de papa y material de mejoramiento utilizando 3.586 marcadores de polimorfismo de nucleótido único (SNP).

**Tablas**

**Tabla Suplementaria S1**. Lista de accesiones de genotipos de papa de la Colección Central Colombiana (CCC) y material de mejoramiento analizados en este estudio.

**Tabla suplementaria S2**. Matriz de genotipos que incluyó los 3.586 marcadores polimórficos de nucleótido único (SNP) identificados y filtrados para 1.291 muestras de papa de la Colección Central Colombiana (CCC) y del material de mejoramiento analizado en este estudio.

**Archivos**.

**Archivo S1**. El manuscrito traducido al español.
