# Supplementary figures and images for: Defining a diverse core collection of the Colombian Central Collection of potatoes: a tool to advance research and breeding
Source: Front Plant Sci. 2023 Apr 26;14:1046400. doi: 10.3389/fpls.2023.1046400 (PMC10173156; doi:10.3389/fpls.2023.1046400)

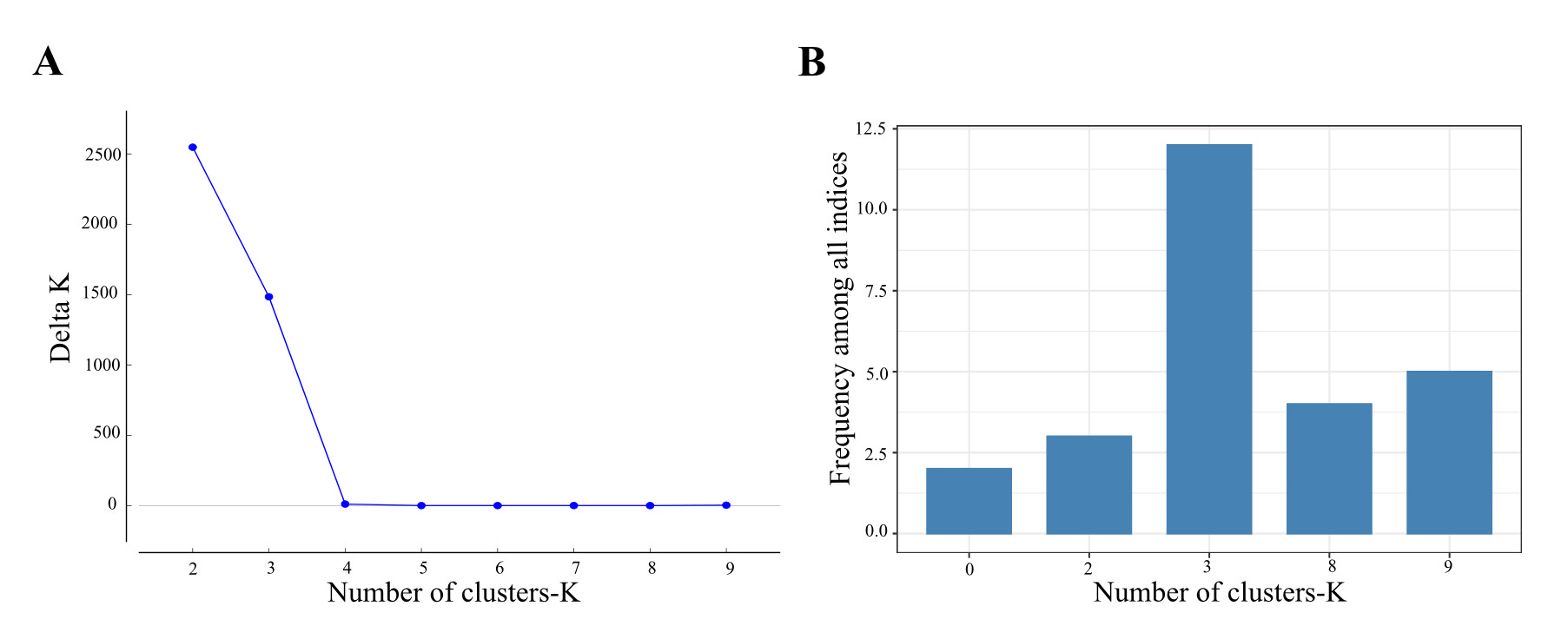

Supplement: Supplementary Figure 1 — Number of genetic clusters suggested by (A) STRUCTURE Harvester (B) and NBClust R-Package test for 1,291 genotyped samples of the Colombian Central Collection (CCC) of potatoes and breeding material using 3,586 Single Nucleotide Polymorphism (SNP) markers. [file Image_1.tif]
